# A task level fusion autonomous switching mechanism

**DOI:** 10.1371/journal.pone.0287791

**Published:** 2023-11-13

**Authors:** Bingyu Lv, Xianchang Wang, Rui Zhang

**Affiliations:** 1 College of Computer Science and Technology, Jilin University, Changchun, Jilin, China; 2 Key Laboratory of Symbolic Computation and Knowledge Engineering, Jilin University, Changchun, Jilin, China; 3 Chengdu Kestrel Artificial Intelligence Institute, Sichuan, China; Guangdong University of Technology, CHINA

## Abstract

Positioning technology is an important component of environmental perception. It is also the basis for autonomous decision-making and motion control of firefighting robots. However, some issues such as positioning in indoor scenarios still remain inherent challenges. The positioning accuracy of the fire emergency reaction dispatching (FERD) system is far from adequate to support some applications for firefighting and rescue in indoor scenarios with multiple obstacles. To solve this problem, this paper proposes a fusion module based on the Blackboard architecture. This module aims to improve the positioning accuracy of a single sensor of the unmanned vehicles within the FERD system. To reduce the risk of autonomous decision-making of the unmanned vehicles, this module uses a comprehensive manner of multiple channels to complement or correct the positioning of the firefighting robots. Specifically, this module has been developed to fusion a variety of relevant processes for precise positioning. This process mainly includes six strategies. These strategies are the denoising, spatial alignment, confidence degree update, observation filtering, data fusion, and fusion decision. These strategies merge with the current scenarios-related parameter data, empirical data on sensor errors, and information to form a series of norms. This paper then proceeds to gain experience data with the confidence degree, error of different sensors, and timeliness of this module by training in an indoor scenario with multiple obstacles. This process is from data of multiple sensors (bottom-level) to control decisions knowledge-based (up-level). This process can obtain globally optimal positioning results. Finally, this paper evaluates the performance of this fusion module for the FERD system. The experimental results show that this fusion module can effectively improve positioning accuracy in an indoor scenario with multiple obstacles. Code is available at https://github.com/lvbingyu-zeze/gopath/tree/master.

## Introduction

With the rapid development of robots, they are used in various fields [1]. Researchers have focused on firefighting robots that have reduced casualties among rescuers. When a fire occurs in an indoor environment, the firefighting robot can adjust to the dynamic indoor environment in order to achieve a safe rescue or accurate firefighting [2, 3]. Accurate positioning is very important for firefighting robots in order to be able to do rescue work accurately [4]. In indoor scenarios where firefighting robots perform tasks, GPS signals are often unavailable [5]. The current indoor positioning methods have many methods, including ultra wide band (UWB), inertial measurement unit (IMU), infrared depth sensor (IDS), camera [4, 6–11]. UWB has high temporal resolution but is susceptible to indoor environments [4, 6]. The IMU is highly accurate, but there is a cumulative error [7]. IDS is easily detected at close range but has limited detection distance, high noise, and low accuracy [8, 9]. Camera accuracy is great, but the real-time performance is poor and vulnerable to light conditions [10, 11]. These sensors can be used to their advantage in different usage scenarios and are difficult to replace. Therefore, the problem of indoor positioning is still inherent.

The single sensor has limited information and is vulnerable to environmental influences. As a result, firefighting robots usually carry a large number of different types of sensors. The information acquired by multiple sensors may complement or contradict each other [12, 13]. The information acquired by each sensor has to be processed separately, increasing the workload. Therefore, multi-sensor fusion is necessary to process the information. Information fusion can improve information reliability, robustness, and utilization [4, 5, 7]. The existing research on multi-sensor fusion framework mainly focuses on the response to specific situations [7, 14, 15]. Sara et al. [14] proposed a three-level positioning framework based on additional visual sensors. The framework obtains the absolute position of the vehicle by tracking landmarks and also improves the GNSS measurement method. Li et al. [7] designed an indoor positioning method based on multiple sensors. They also constructed an extended Kalman filtering (EKF) algorithm framework. Dasgupta et al. [15] proposed sensor fusion software for autonomous vehicles and other autonomous robots to achieve precise positioning of autonomous robots. However, multi-sensor fusion is susceptible to environmental interference, leading to measurement errors and uncertainties. Therefore, facing a large amount of uncertain data, multiple domain experts are needed to process this data. The blackboard architecture has proven to be a very useful and flexible mechanism [16, 17]. In this paper, a fusion module in FERD system is proposed based on the Blackboard Architecture. The overall goal is achieved by quickly and autonomously switching different strategies for multiple unmanned vehicles. The position of unmanned vehicles is supplemented or modified by multiple channels. These strategies can reduce the impact of inaccurate positioning in an open environment, also reduce the risk of autonomous decision-making of each unmanned vehicle. Then, this fusion module in FERD system is realized in the task of firefighting. Finally, the performance of the proposed fusion module is evaluated. The main contributions of this paper are summarized as follows.

- In this paper, a fusion module is designed and added to fire emergency reaction dispatching (FERD) system. This fusion module is used for accurately positioning fire equipment by multiple sensors. All sensors send their measured position information as primary data to this module. The position information of the real physical position of each fire equipment is used as auxiliary data. This module has developed a variety of fusion-related processing for precise positioning. These processes mainly include six strategies. They are the denoising, spatial alignment, confidence degree update, observation filtering, data fusion, and fusion decision. Compared to a single sensor, this paper proposed module is effective and scalable.
- Multiple unmanned vehicles perform firefighting tasks and collect data in an indoor scenario with multiple obstacles. This demonstrates the effectiveness of the module in locating sensors in the field. This module merges with the current scenarios-related parameter data, empirical data on sensor errors, and information to form a series of norms. This paper then proceeds to gain experience data with the confidence degree, error of different sensors, and timeliness of this module by training in an indoor scenario with multiple obstacles. This process is from data of multiple sensors (bottom-level) to control decisions knowledge-based (up-level). This process can obtain globally optimal positioning results.

The remainder of this paper is organized as follows. Section RELATED WORKS introduces the related to the work of this paper. Section PRELIMINARIES introduces a few concepts and reviews previous works. Then, a few basic terms are provided that will be used in later sections. Section PROPOSED MODULE, this section emphasizes proposed module. Section RESULTS analyzes the errors of different sensors in detail. Then, this section introduces how to implement the fusion module based on Blackboard Architecture. Finally, this section gives the experimental results and analysis. Section DISCUSSION discusses the advantages and disadvantages of the proposed fusion module. Section CONCLUSION summarizes the significance of the fusion module and discusses future work.

## Related works

This paper presents a novel and comprehensive sensor fusion scheme. The focus of this work is on the fusion method and the switching of fusion methods.

### Kalman filtering

Some existing works with us have related ideas to Kalman Filtering as one of the data fusion methods. For the indoor positioning problem of miniature tracked robots, Li et al. [7] designed an indoor positioning method. This method is based on Bluetooth, a gyroscope, an accelerometer, a magnetometer, and other sensors. This method takes an inertial navigation system (INS) as the core, Bluetooth AOA positioning base station as the position observation, and magnetometer as the heading angle observation. They also constructed an EKF algorithm framework. This EKF is based on error states. Kim and Lee [18]proposed the EKF algorithm. This algorithm is a combination of a Camera, GPS, and sensor of in-vehicle for the precise positioning of vehicles. This algorithm combines multiple decisions made by different sensors and finally makes a fusion decision. Luo et al. [19] proposed a joint Kalman filter to fuse the positioning information. This information is the combined localization subsystem. This method provides critical position information for achieving high accuracy and efficient missions. The work of this paper determines whether to fuse the data from multiple sensors through confidence degree and error size. Then this work of this paper uses the Kalman filter to fuse the data. In this way, the proposed approach provides critical position information during operation. The proposed approach improves the positioning accuracy and efficient tasks of FERD system.

### Multi-sensor fusion based blackboard architecture

The proposed fusion module combines the norms of data fusion and error parameters of multiple sensors with the Blackboard Architecture. Benjamin et al. [20] proposed that the design of the transmission trigger and estimator should be closely combined. The intelligent trigger mechanism of sensors can predict future information about these sensors. The purpose of this method further reduces the communication demand. Girraj et al. [21] optimized and evaluated thresholds of fusion norms. They proposed an iterative algorithm to calculate the individual optimal threshold of fusion norms. Based on the necessary and sufficient conditions of the optimal sensor norm, Liao et al. [22] derived a set of norms for sensors. These norms are significantly superior to the traditional iterative search algorithm. These norms are applied to multi-sensor decision fusion problems with high-dimensional sensors. Yang et al. [23] proposes a sensor fusion algorithm. They create redundancy, by using multiple sensors to measure the same physical variables. This algorithm is used to provide robust estimation with correct sensor information. These controllers reduce estimation errors and communication channel noise. However, multi-sensor fusion is susceptible to environmental interference, leading to measurement errors and uncertainties. The difference between the method proposed in this paper and these methods is that the accuracy of multiple sensors is considered, and some norms about accuracy are designed for fusion. In different environments, our method can filter the measurement accuracy of multiple sensors and switch the corresponding strategies about fusion. Since norms of accuracy require a combination of parametric and empirical data related to the current environment, firefighting robots need to be trained to obtain these data. These training values can be used as empirical values for FERD system to perform tasks. In this way, the method proposed in this paper is more likely to improve the correctness of decisions and reactions during the operation of FERD system.

## Preliminaries

In this section, the Blackboard System (BBS) and General Blackboard Open Source (GBBopen) are first introduced. Then, previous work and some concepts are introduced. Finally, the basic concepts covered in this paper are introduced in detail.

### Blackboard system and generic blackboard open source

**(1) Blackboard system**. BBS is an artificial intelligence technique based on knowledge bases and collaborative processing, which is a distributed computing model. It is widely used in complex problem solving, for example, in the field of decision support. It enables effective collaborative processing of multiple specialized modules to achieve more intelligent and efficient problem solving. The operation of the BBS is driven by events. It comprises a **knowledge source (KS)**, **blackboard (BB)**, and **control shell (CS)**. In the BBS, a KS is similar to an expert. Each KS has its own expertise and capabilities. These KSs work together through a central data structure (BB) to solve a complex problem. BB is a shared data structure. Specifically, the information on the BB can be the description of the problem, the solution of the problem, etc. All KSs can read and write the information on BB to achieve the goal of solving problems together. A KS can write the problem or the solution on BB. KS can also read the information on the BB and process and solve the problem according to its knowledge and abilities. CS is an important part of the BBS. It is responsible for monitoring the information on the BB as well as coordinating and controlling the interaction and decision-making process between the various KSs. When new information appears on the BB, the CS can decide which KSs can read it and how to deliver it to other KSs according to predefined norms and strategies.

**(2) Generic blackboard open source**. GBBopen is an open-source blackboard system. It can be used to build various types of intelligent systems and applications. GBBopen provides a range of tools and supports multiple programming languages and operating systems. It can run on different platforms and be extended and customized on demand. Developers are able to quickly build and customize their own blackboard systems, implementing various types of intelligent applications and systems. In this paper, GBBopen is developed and implemented in the common list processing (Common Lisp) language environment. An expert system based on the norm-base developed by Clips (https://www.clipsrules.net) is a data-driven program. Unlike Clips, GBBopen does not require a data abstraction design; therefore, the implementation of a function may be completed quickly during development.

In GBBopen, the unit class is the base class for all classes defined. The properties of KS are ‘trigger-events’, ‘precondition-function’ and ‘execution-function’. Each knowledge source activation (KSA) is an instance of a KS. The attributes of KS and KSA as follows: *ks*_*i*_ = {*event*, *pre*, *exe*, *rating*}; *ksa*_*i*_ = {*rating*, *exe*}. The running process of the CS is as follows: First, enter a series of KS. When an event occurs, the CS determines whether it is consistent with the *ks*_*i*_.*event*. If they are consistent, the state reads the data of agents from BB and the function *ks*_*k*_.*pre* is executed. The variable value of *ks*_*i*_.*pre* is provided by state. If the return value of the function *ks*_*i*_.*pre* is true, then *ks*_*i*_ is triggered and generates an instance *ksa*_*i*_, which stores a list of ‘pending-ksas’. Then, *ksa*_*k*_.*exe* in ‘pending-ksas’ with the highest level is executed. The data of the agents on BB are changed. When certain conditions are met, the GBBopen ends its operation.

### Autonomous switching of task-level strategies

In this subsection, we have previously developed a framework for ASTS [24]. ASTS can automatically switch strategies according to the different scenarios to react to various emergencies.

#### Implementation for ASTS

ASTS is described in the form of tuples, a set of norms *N*, state *S*, and event *E*.

$$\begin{array}{c} ASTS=<N,S,E> \end{array}$$ (1)

- **Norm *N***. *N* = {*r*_1_, *r*_2_, …} represents a set of norms, which are the mapping relationships between predefined conditions and agent behavior and are the key to the effective operation of ASTS. Norm *r*_*i*_ is expressed as *r*_*i*_ = {*Tri*_*i*_, *Act*_*i*_, *Exp*_*i*_}.
  - *Tri*_*i*_ is the trigger condition and is used to determine if the associated actions can be performed. *Tri*_*i*_ consists of a series of logical judgment, which can be expressed as ${Tri}_{i}=\{tri_{i_{1}},tri_{i_{2}},\ldots \}$.
  - *Act*_*i*_ is the execution action. *Act*_*i*_ consists of a series of methods or actions, which can be expressed as ${Act}_{i}=\{act_{i_{1}},act_{i_{2}},\ldots \}$.
  - *Exp*_*i*_ is the expected result to verify the result of *Act*_*i*_. *Exp*_*i*_ consists of a series of expected conditions, which can be expressed as ${Exp}_{i}=\{exp_{i_{1}},exp_{i_{2}},\ldots \}$.
- **State *S***. The data are read by State *S*. State *S* represents a set of instantaneous states denoted by *S* = {*s*_1_, *s*_2_, …}, which is arranged in chronological order. The instantaneous state *s*_*i*_ consists of a series of expressions, which are expressed as $s_{i}=\{s_{i_{1}},s_{i_{2}},\ldots \}$.
- **Event *E***. *E* = {*e*_1_, *e*_2_, …, *e*_*i*_} represents a set of instantaneous events sorted in chronological order. The instantaneous event *e*_*i*_ consists of a series of sub-events, which are expressed as $e_{i}=\{e_{i_{1}},e_{i_{2}},\ldots \}$. When an event *e*_*i*_ occurs, the instantaneous state *s*_*j*_ is read to determine the *Tri*_*k*_ of *r*_*k*_.

#### Operation process of ASTS

**Definition 1. Norm, Event, and State**. Eq (1) describes the components of ASTS. For example, a logistics truck named Tom starts work at 1o o’clock every day. Tom must follow the norms listed in the Table 1. According to norms *r*_*a*_ and *r*_*b*_, Tom maintains a safe distance W from the object in front of it (set at 0.5m). If W is greater than 0.5 m and the power is greater than 30% when *e*_1_ occurs, then Tom moves forward. To verify the *Act*_*a*_ result of the norm *r*_*a*_, we expect W to be equal to 0.5 m. If W is less than 0.5 m and the power is less than 30% when *e*_1_ occurs, Tom stops. The instantaneous events and states are listed in Table 2.

**Table 1:** Example of norm.

Norm	Trigger	Action	Expectation
*r*_*a*_	*W* > 0.5 m, *power* > 30%	Tom forward	*W* = 0.5 m
*r*_*b*_	*W* < 0.5 m, *power* > 30%	Tom stop	*W* = 0.5 m
*r*_*c*_	*W* < 0.25 m, *power* > 30%	Tom backward	*W* > 0.25 m

**Table 2:** Recording of instantaneous events and states.

Time	Instantaneous event	Instantaneous state
9:00	Logistics Truck open Subscribe to Tom’s position	*W* = 1.6 m, *power* = 40%
9:30	Subscribe to Tom’s position	*W* = 1 m, *power* = 34%
9:40	Subscribe to Tom’s position	*W* = 0.4 m, *power* = 31%
9:50	Subscribe to Tom’s position	*W* = 0.3 m, *power* = 29%

**Definition 2. Triggered norm**. When *e*_*i*_ occurs, the instantaneous state *s*_*i*_ is read. If all logical judgment expressions in *Tri*_*x*_ are satisfied, then Norm *r*_*x*_ is denoted as the ‘triggered norm’ at instantaneous state *s*_*i*_. For example, an instantaneous event *e*_1_ occurs at 9:00. *Tri*_*a*_ is satisfied at the instantaneous state *s*_1_; then Tom starts moving forward. Norm *r*_*a*_ is denoted as the ‘triggered norm’.

**Definition 3. State transition**. When *e*_*i*_ occurs, norm *r*_*x*_ is triggered at *s*_*i*_, and *Act*_*x*_ is executed. This process causes the instantaneous state to change from *s*_*i*_ to *s*_*j*_, which can be expressed as:

$$\begin{array}{c} s_{i}\overset{r_{x}}{\to }s_{j} \end{array}$$ (2)

Where the notation ‘→’ denotes an instantaneous state changed from *s*_*i*_ to *s*_*j*_ as a result of triggering *r*_*x*_. If the instantaneous state changed from *s*_1_ to *s*_2_ as a result of triggering *r*_*a*_. The instantaneous state changes from *s*_2_ to *s*_3_ as a result of triggering *r*_*a*_. These processes are expressed in Formula (3).

$$\begin{array}{c} s_{1}\overset{r_{a}}{\to }s_{2}\overset{r_{a}}{\to }s_{3} \end{array}$$ (3)

**Definition 4. Strategy**. Norms are the key to the effective operation of multiple agent systems and are a predefined mapping relationship between conditions and behaviors. A strategy is a plan of action based on the current environment and goals. It is used to guide the agent’s decisions and behaviors in a specific domain and helps the agent achieve its goals in that domain. A strategy consists of a set of action plans, and norms can be used as part of developing a strategy. For example, Norms *r*_*a*_ and *r*_*b*_ form a goal-following strategy in Table 1. Norm *r*_*c*_ forms an obstacle-avoidance strategy.

**Definition 5. Switch norms or strategies**. When *e*_*i*_ occurs, norm *r*_*x*_ is triggered at *s*_*i*_. Then, *e*_*j*_ occurs and *r*_*y*_ is triggered at *s*_*j*_. The norm switches from *r*_*x*_ to *r*_*y*_. Multiple switching norms accumulate when one norm is switched to another. For example, when *e*_1_ occurs, Norm *r*_*a*_ is triggered at *s*_1_. Then, *e*_2_ occurs, and the norm *r*_*a*_ is triggered at *s*_2_. Finally, *e*_3_ occurs, and the norm *r*_*b*_ is triggered at *s*_3_. The norm switches from *r*_*a*_ to *r*_*a*_ and eventually to *r*_*b*_.

Based on the above description, this study summarises the pseudocode of ASTS as follows:

i. Input *N*, *S*, *E*, *T*;
ii. Initialisation: *Path* = *ϕ*, *Res*_*Act*_ = *ϕ*, *Res*_*Exp*_ = *ϕ*, *i* = 0, *j* = 0, *k* = 0.
iii. If an instantaneous event *e*_*i*_ ∈ *E* occurs, the ASTS data are read by the transient state *s*_*j*_ ∈ *S*.
iv. Judge *Tri*_*k*_ of Norm *N*.
v. If *Tri*_*k*_ = *True*, Norm *r*_*k*_ is triggered; perform *Act*_*k*_ and judge *Exp*_*k*_.
vi. The results for *Act*_*k*_ and *Exp*_*k*_ are restored as *Res*_*Act*_ and *Res*_*Exp*_, respectively.
vii. *s*_*i*_, *e*_*j*_, *r*_*k*_, *Res*_*Act*_, *Res*_*Exp*_ denote the restored paths.
viii. *j* + +, *i* + +.
ix. Until *i* > *T*, exit ASTS.

### Fire emergency reaction dispatching system

We previously developed a FERD system applied to a fire scenario to demonstrate the autonomy and switchability of ASTS [24]. We designed five strategies and two control modes for the FERD system to enable multiple types of firefighting equipment to switch between multiple strategies and multiple control modes depending on the changing environment. The system is described in detail below.

#### Components of FERD system

The FERD system system is based on the framework of ASTS. The components include a set of tasks *M*, a set of fire equipment *A*, a set of sensors *Q*, and a set of agent norms *N*′. FERD system can be expressed as:

$$\begin{array}{c} FERD=<M,A,Q,N^{\prime }> \end{array}$$ (4)

**Fig 1:**
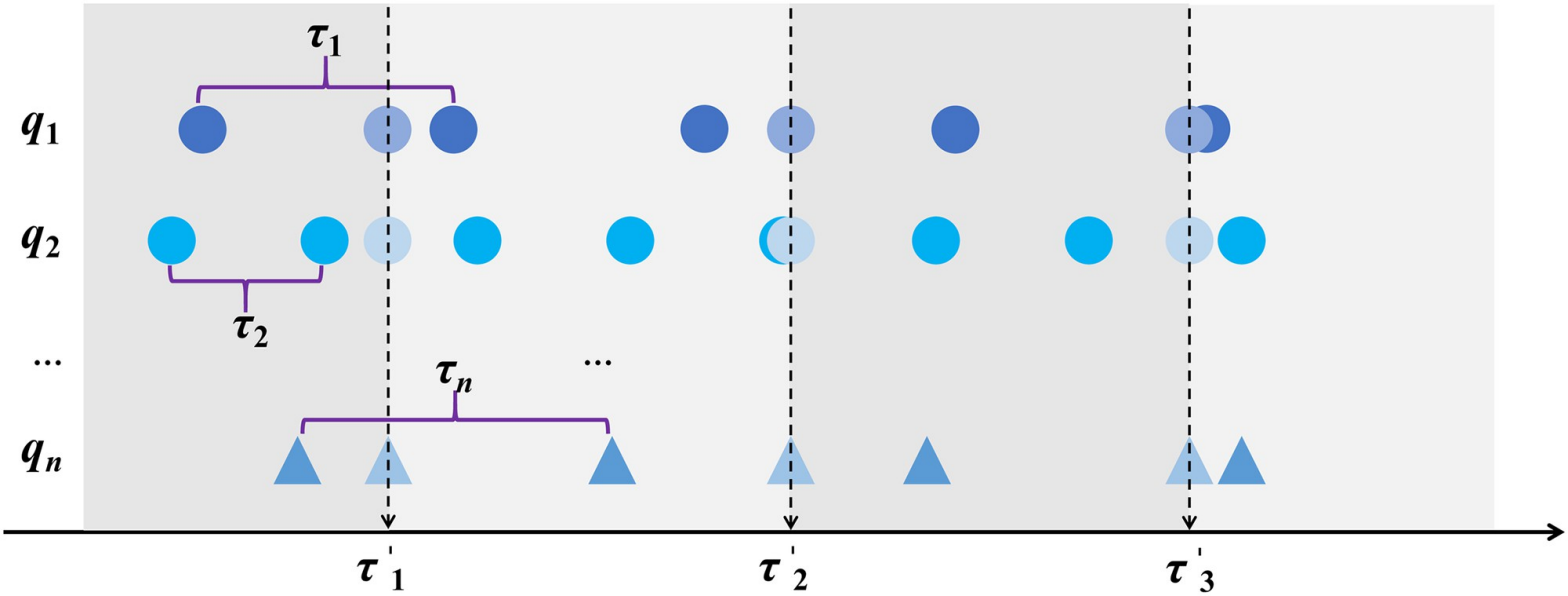
Time alignment in FERD system. The time alignment process of n sensors.

- **Task *M***. The FERD system is applied to handle fire extinguishing tasks, Task *M* = {*m*_1_, *m*_2_, …}. Each task *m*_*i*_ comprises a series of attributes denoted as $m_{i}=\{m_{i_{1}},m_{i_{2}},\ldots \}$.
- **Agent *A***. A certain number of agents is needed to achieve the ultimate goal, which can be expressed as A = {*a*_1_, *a*_2_, …}. *a*_*i*_ denotes the *i*-th fire equipment. *a*_*i*_ = {*u*_*i*1_, *u*_*i*2_, …, *u*_*ik*_} denotes the attribute of *a*_*i*_. *u*_*ik*_ denotes the *k*-th attribute of *a*_*i*_.
- **Sensor *Q***. Each agent carries sensors to sense the environment. Sensor *Q* = {*q*_1_(*τ*_1_), *q*_2_(*τ*_2_), …}, and *q*_*i*_ denotes the *i*-th sensor. The sampling periods of multiple sensors are different. *τ*_*i*_ is the sampling period of sensor *q*_*i*_. The sensor *q*_*i*_ can obtain real values and observed values of the attribute *u*_*jk*_ of the fire equipment *a*_*j*_ during each sampling period *τ*_*i*_. It can be expressed as $q_{i}(\tau_{i})=\{<u_{jk(i,\tau_{i1})}^{true},u_{jk(i,\tau_{i1})}^{false}>,<u_{jk(i,\tau_{i2})}^{true},u_{jk(i,\tau_{i2})}^{false}>,\ldots \}$. $u_{jk(i,\tau_{i1})}^{true}$ denotes real values of sensor *q*_*i*_ for measure attribute *u*_*jk*_ of fire equipment *a*_*j*_ during the first sampling period *τ*_*i*1_. $u_{jk(i,\tau_{i1})}^{false}$ denotes observed values of sensor *q*_*i*_ for measure attribute *u*_*jk*_ of fire equipment *a*_*j*_ during the first sampling period *τ*_*i*1_. These sensors have different sampling periods. At each time step $\tau_{l}^{\prime }$, observed value of each sensor is obtained in FERD system. This process is shown in Fig 1.
- **Agent Norm *N*′**. The norm set *N*′ for the agent, which is denoted by $N\prime =\{r_{1}^{\prime },r_{2}^{\prime },\ldots \}$. Where norm $r_{k}^{\prime }$ is expressed as $r_{k}^{\prime }=\{Tri_{k}^{\prime },Act_{k}^{\prime },Exp_{k}^{\prime }\}$. The internal elements of $Tri_{k}^{\prime }$, $Act_{k}^{\prime }$, $Exp_{k}^{\prime }$ and the internal elements of *Tri*_*k*_, *Act*_*k*_, *Exp*_*k*_ are consistent.

#### Objectives of FERD system

The objectives of FERD system are expressed as Eq (5). Minimize the maximum time for FERD system rescue to complete tasks. And all tasks in *M* are completed.

$$\begin{array}{c} \left\{ \begin{array}{c} \text{min}\;t_{\text{max}},\\ M=\phi . \end{array}\right. \end{array}$$ (5)

#### Constituent of FERD system

The FERD system operates based on the GBBopen platform. In a FERD system, a set of multiple KSs make up a strategy. The CS selects the appropriate KS for the multiple agents to switch. After the strategy is executed, the data on the BB are changed until the fire extinguishing tasks are completed. Certain properties of the agent are obtained from external sensors. The components of the FERD system are described in detail below.

**(1) Blackboard**. In the FERD system, a task and a type of agent are defined as classes, respectively. Each agent is defined as an instance. The attribute value of each agent is either directly or indirectly defined.
**(2) Knowledge Source**. KS is a core component of the FERD system. In GBBopen, the attributes of KS are in correspondence with the Agent Norm *N*′, and the properties of the KS are extended, ‘**postcondition-function**’. The relationship between *N*′ and KS can be formally described as follows: $Tri_{i}^{\prime }=ks_{i}.event$ and *ks*_*k*_.*pre*, $Act_{i}^{\prime }=ks_{i}.exe$, $Exp_{i}^{\prime }=ks_{i}.post$. KSs are divided into two submodules: the **Switch Module** and **Strategy Module**.
  **1) Classification of Switch Module**. The agent can switch between the strategies based on multiple switch modules. The control modes are divided into **Automatic Mode** and **Command Mode**. Multiple agents can switch strategies based on their own identification or environmental identification, or based on user requirements or external guidance.
  **2) Classification of Strategy Module**. Different strategies are generated from the agent state and the collected environmental information. These mainly include the **Following Strategy**, **Motor Strategy**, **Evolution Strategy**, **Obstacle Avoidance Strategy**, and **Mobilization Strategy**.
    **a. Movement Strategy**. The movement strategies are divided into **Forward**, **Stop**, and **Fallback**.
    **b. Following Strategy**.**Trajectory Following** and **Goal Following** are developed for agents to arrive at fire scenarios rapidly.
    **c. Mobilization Strategy**. In different scenarios, multiple agents can select **Formation**, **Leave Team**, or **Return To Team**.
    **d. Obstacle Avoidance Strategy**. Multiple agents can select **Virtual Circle**, **Virtual Right Triangle**, and **Virtual Isosceles Triangle**.
    **e. Evolutionary Strategy**. The FERD system provides two evolutionary strategies for autonomously evolving norms: **Crossover** and **Other Method**. In Crossover, the expectation of one mutated norm enrich the trigger of another mutated norm.
**(3) Control Shell**. In the FERD system, after *ksa*_*k*_.*exe* is executed, *ksa*_*k*_.*post* is executed. The CS triggers various norms based on event changes to achieve fast switching strategies.

### Coordinate transform

Because the three-dimensional coordinate cannot directly transform the two-dimensional coordinate, it is necessary to obtain the mapping relationship of each coordinate. The transformation between the world coordinate and the camera coordinate is through rigid body transformation. The transformation between the camera coordinate and the image coordinate is through perspective projection. The transformation between the image coordinate and the pixel coordinate is through affine transformation. The transforming formula between a point in the world coordinate and a point in pixel coordinate can be expressed as:

$$\begin{array}{cc} Z_{c}*[\begin{array}{c} u\\ v\\ 1 \end{array}] & =K_{3\times 4}*RT_{4\times 4}*[\begin{array}{c} X_{w}\\ Y_{w}\\ Z_{w}\\ 1 \end{array}]\\ K_{3\times 4} & =[\begin{array}{cccc} f_{x} & 0 & u_{0} & 0\\ 0 & f_{y} & v_{0} & 0\\ 0 & 0 & 1 & 0 \end{array}]\\ RT_{4\times 4} & =[\begin{array}{cc} R_{3\times 3} & t_{3\times 1}\\ 0_{1\times 3} & 1_{1\times 1} \end{array}] \end{array}$$ (6)

Among them, *Z*_*c*_ indicates the distance between the camera and the point in the world coordinate. This distance can be obtained in other ways. *K*_3×4_ indicates internal reference matrix. *RT*_4×4_ represents external reference matrix. *f*_*x*_, *f*_*y*_ indicate focal length. Generally, they are equal. *R*_3×3_ represents rotation matrix, which describes the direction between the coordinate axis of a world coordinate and the coordinate axis of the camera coordinate. *t*_3×1_ indicates translation matrix, which describes the position of the origin of a world coordinate in the camera coordinate.

### Camera calibration

The calibration method of Zhengyou Zhang is to fix the world coordinate system on the chessboard. This world coordinate system is defined in advance. Capture an image of the chessboard through the camera. The pixel coordinates (*u*, *v*) of each corner point are obtained using the image detection algorithm. The corner point is the connection point of the contour line of the object. The coordinate of each corner point (*X*_*w*_, *Y*_*w*_, *Z*_*w*_=0) is calculated in the world coordinate system. Through Eq 6, the mapping relationship between the world coordinate and pixel coordinate is calculated. The internal parameter matrix (e.g. *K*_3×4_) and external parameter matrix (e.g. *R*_3×3_, *t*_3×1_) are obtained.

### Monocular measurement

The measurement methods based on monocular vision can be divided into multiple categories. One of the generalizations is the ratio-based approach. The principle is that the distance is inversely proportional to the image size. The calculation formula is:

$$\begin{array}{c} D^{\prime }=\frac{W\times f}{P} \end{array}$$ (7)

Among them, *f* represents Focal length; *P* represents Pixel width of an object, pixel; *W* expresses Actual width of an object, m; *D*′ represents that unknown distance from an object to a camera, m.

### Kalman filter based confidence degree

Kalman Filter is a data fusion algorithm. This algorithm fuses the measured values of different sensors with the same object. The measurement noise satisfies a normal distribution. Finally, a more accurate value is obtained. To study the consistency of sensor data, the concept of confidence degree is introduced here.

Suppose there exist n independent sensors. There are *q*_1_, *q*_2_, …, *q*_*n*_. The fusion process of the Kalman Filter based on confidence degree is as follows.

$$\begin{array}{cc} q_{0_{t}} & =w_{1_{t}}*q_{1_{t}}+w_{1_{t}}*q_{1_{t}}+\cdots +w_{n_{t}}*q_{n_{t}}\\ {\hat{x}}_{0_{t}} & =w_{1_{t}}*x_{1_{t}}^{\prime }+w_{1_{t}}*x_{1_{t}}^{\prime }+\cdots +w_{n_{t}}*x_{n_{t}}^{\prime }\\ \sigma_{0_{t}} & =\sigma_{1_{t}}+\sigma_{1_{t}}+\cdots +\sigma_{n_{t}}\\ R_{0} & =R_{1}+R_{2}+\cdots +R_{n}\\ K^{2} & =\frac{\sigma_{0_{t}}^{2}}{\sigma_{0_{t}}^{2}+R_{0}^{2}}\\ q_{fuse_{t}} & ={\hat{x}}_{0_{t}}+K*\left(q_{0_{t}}-{\hat{x}}_{0_{t}}\right)\\ \sigma_{fuse_{t}} & =\sqrt{\left(1-K\right)*\sigma_{fuse_{t-1}}}\\ x_{i_{t}} & =F_{i_{t}}*x_{i_{t-1}}+B_{i_{t}}*\mu_{i_{t}}\;\left(i=1,2,\cdots ,n\right) \end{array}$$ (8)

Among them, *K* indicates Kalman gain. The degree of change in uncertainty after each fusion of data. A lower Kalman gain helps to improve the accuracy of fusion and vice versa. *R*_*i*_ represents measurement error of the *i*-th sensor. *R*_0_ represents measurement error of all sensors. $q_{i_{t}}$ indicates observation data of the *i*-th sensor. $x_{i_{t}}$ indicates equation of state of the *i*-th sensor. $x_{i_{t}}^{\prime }$ indicates the optimal estimate of the *i*-th sensor. It is obtained by classical Kalman filtering. At time *t*, $F_{i_{t}}$ represents state transfer matrix of the *i*-th sensor. $B_{i_{t}}$ indicates control matrix of the *i*-th sensor. $\mu_{i_{t}}$ represents control vector of the *i*-th sensor. $\sigma_{i_{t}}$ indicates standard deviation of the *i*-th sensor. $w_{i_{t}}$ indicates confidence degree of the *i*-th sensor. $q_{0_{t}}$ represents observations based confidence degree. ${\hat{x}}_{0_{t}}$ indicates predicted values based confidence degree. $\sigma_{0_{t}}$ indicates standard deviation based confidence degree. $q_{fuse_{t}}$ represents fusion results of multiple sensors. $\sigma_{fuse_{t}}$ indicates standard deviation of multiple sensors.

As can be seen from Eq 8, the effective fusion of multiple sensors is achieved. This process adjusts the confidence degree of each sensor in real time. When the observed values of a sensor is not accurate, the algorithm has a certain fault-tolerant ability. This algorithm affects the overall performance of multi-sensor fusion. In this paper, the Kalman Filter based confidence degree has the ability to be adaptive.

## Proposed module

In this paper, for the phenomenon of inaccurate observed values of multiple sensors, Fusion Module is added in FERD system. This section provides a detailed description of the supplement or correct of a single sensor.

### Fusion module of FERD system

This study added the fusion module in FERD system. KS consists of three sub-modules. They are the control module, execution module, and fusion module.

### KS of fusion module

**Fig 2:**
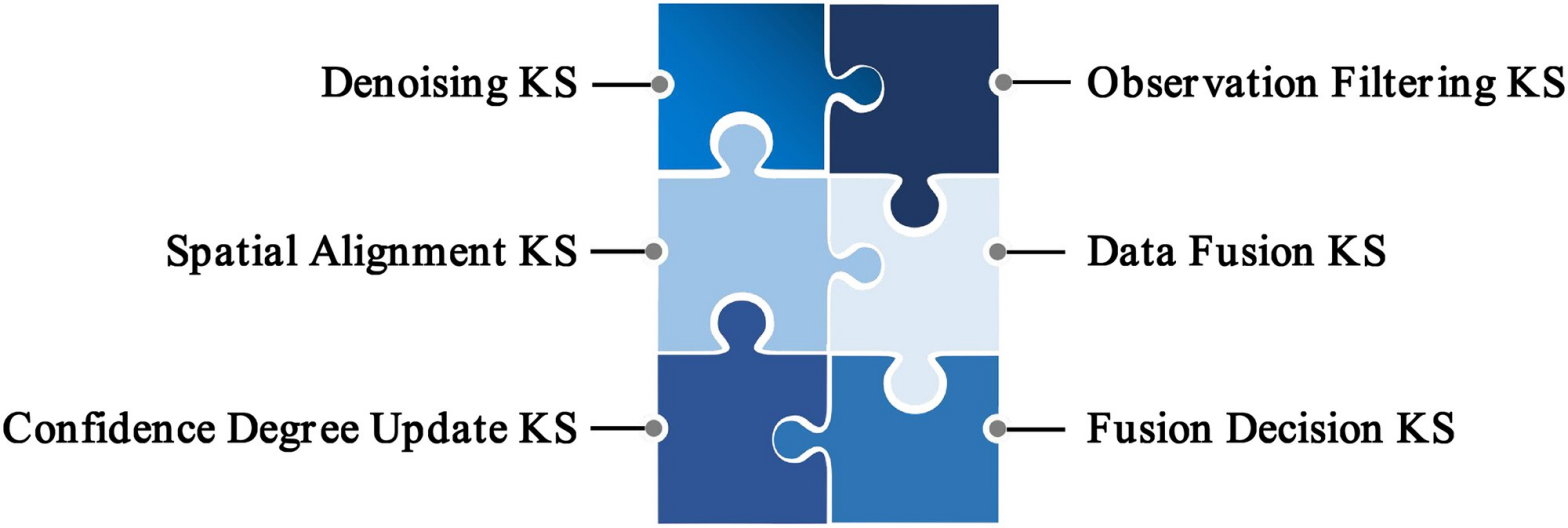
Fusion module in FERD system. The Fusion Module include denoising, spatial alignment, confidence degree update, observation filtering, data fusion, and fusion decision.

This subsection provides a detailed description of Fusion Module. Experts have developed a series of fusion strategies for the fusion of multiple sensors. As shown in Fig 2.

#### Denoising

Interference values exist for every sensor. Interference values are values that describes the scenario inaccurately. In this paper, interference values are collectively referred to as random errors. Then the observed values contains a large number of random errors. The random errors not only increase the computer memory and computation overhead but also increase the computation error. So observed values of each sensor need denoising. The purpose of denoising for sensor *q*_*i*_ is to reduce the Standard Deviation *σ*_*i*_.

#### Spatial alignment

The observed value of each sensor in the same platform using different coordinate systems. The observed value of different sensors involve different coordinate systems. Therefore, observed value must be converted into data in the same coordinate system before fusion. In fact, this process is to calculate the transformation relationship between coordinate systems. The system converts the attribute *u*_*jk*_ of the fire equipment *a*_*j*_ into a uniform type of sampled data from sensor *q*_*i*_ by Spatially Aligning KS. In this paper, the transformation matrix of sensor *q*_*i*_ is set to *trans*_*i*_. The observation for each sampling period $\tau_{l}^{\prime }$ after spatial alignment is $u_{jk(i,\tau_{l}^{\prime })}^{false}*trans_{i}$. The real value of the sampling period $\tau_{l}^{\prime }$ after spatial alignment is $u_{jk(i,\tau_{l}^{\prime })}^{true}*trans_{i}$.

#### Confidence degree update KS

The contribution of each sensor to data fusion is also different. Therefore, this paper proposes the concept of confidence degree. The fusion contribution of each sensor is defined by the confidence degree. For example, sensor *q*_*m*_ and *q*_*n*_ are both sensors that make observations of *u*_*jk*_. In each sampling period, their confidence degrees are updated by this KS. Their confidence degrees are denoted by *w*_*mg*_ and *w*_*ng*_, respectively. Where $g=1,2,\ldots ,t_{\text{max}}$.

#### Observation filtering

The error of observed values from each sensor is different. Therefore, the concept of error threshold is proposed in this paper. It serves as a precondition for fusion. The error of observed values of sensor *q*_*i*_ on *u*_*jk*_ in each sampling period is expressed as follows.

$$\begin{array}{c} \varepsilon_{mg}=|u_{jk(m,\tau_{g}^{\prime })}^{false}*trans_{m}-u_{jk(m,\tau_{g}^{\prime })}^{true}*trans_{m}| \end{array}$$ (9)

For example, sensor *q*_*m*_ and *q*_*n*_ measure for *u*_*jk*_. Then, this paper calculates the error between observed values and real values. Their errors are denoted as *ε*_*mg*_ and *ε*_*ng*_, respectively. Select their error thresholds by this KS. Their error thresholds are denoted as *ε*_*m*_ and *ε*_*n*_, respectively. Finally, observed values of two sensors in each sampling period $\tau_{g}^{\prime }$ are compared with the corresponding error thresholds separately. The filtering operation is performed mainly for Data Fusion KS.

#### Data fusion

This KS is a process of multi-level, multi-spatial information complement and optimal combination processing by multiple sensors. In this process, multiple sources of data are fully utilized for rationalization and use. This not only takes advantage of multiple sensors operating in concert with each other but also integrates data from other information sources. This improves the intelligence of the whole system, as well as the accuracy and comprehensiveness of the information, and reduces the uncertainty of the information. This KS combines observations from multiple sensors into one data. This operation makes the measured values more accurate.

This paper assumes that there are Γ different fusion algorithms in this module. The observed values of sensor *q*_*m*_ and *q*_*n*_ for the same attributes (*u*_*jk*_) are fused. At this point, the observed values from both sensors have been denoised and spatially aligned. During each sampling period $\tau_{g}^{\prime }$, FERD system can obtain observed values of all sensors. ∃*ε*_*mg*_ ≤ *ε*_*m*_. Where $g=1,2,\ldots ,t_{\text{max}}$.

By the fusion algorithm *γ* ∈ Γ, the result of Data Fusion KS during each sampling period $\tau_{g}^{\prime }$ can be expressed as $u_{jk(\tau_{g}^{\prime })}^{fuse_{\gamma }}$. Then,

$$\begin{array}{c} u_{jk(\tau_{g}^{\prime })}^{fuse_{\gamma }}=\left\{\left(w_{mg}*u_{jk(m,\tau_{g}^{\prime })}^{false}*trans_{m}\right)⦾\left(w_{ng}*u_{jk(n,\tau_{g}^{\prime })}^{false}*trans_{n}\right)\right\} \end{array}$$ (10)

Among them, the symbol ‘⦾’ is a symbol for data fusion, which indicates that two observed values are fused. The real values of the same property for the same object is the same for different sensors. The real values of the same property of the same object in each sampling period $\tau_{l}^{\prime }$ can be expressed as $u_{jk(\tau_{g}^{\prime })}^{true_{\gamma }}$.

#### Fusion decision

For the results of different data fusion algorithms, the role of this KS is to select the appropriate fusion algorithm *γ*. This algorithm is a fusion algorithm that is the closest to the real values. That is:

$$\begin{array}{c} \text{min}\;\sum_{g=1}^{t_{\text{max}}}\;|u_{jk(\tau_{g}^{\prime })}^{{fuse}_{\gamma }}-u_{jk(\tau_{g}^{\prime })}^{{true}_{\gamma }}| \end{array}$$ (11)

Where, *j* = 1, 2, …, *x*; *k* = 1, 2, …, *y*′; *γ* ∈ Γ.

## Results

This experiment of performing firefighting tasks in an indoor scenario with multiple obstacles is a specific application scenario of this fusion module proposed. FERD system can use multiple sensors to detect unmanned vehicles during firefighting tasks. To verify the effectiveness of Fusion Module, three sensors are used to assist in the firefighting tasks. This section first introduces experiment design. Then, the experimental steps are described in detail. Subsequently, the experimental results are given. Finally, the effectiveness is further verified by visual analysis.

### Experiment design

In this subsection, the experiment design is first described. And the details are described. Then, the limitations are considered and analyzed. Finally, evaluation criteria are given to verify the effectiveness of this fusion module. Table 3 summarizes the symbols commonly used in this experiment.

**Table 3:** Symbol notations.

Symbol	Description
FER	Fusion Efficiency Rate
SD	Standard Deviation
ED	Euclidean Distance
MSE	Mean Square Error
*num*	The ordinal number of task *m*_*i*_, denoted as *num*_*i*_.
*mp*	The position of task *m*_*i*_, denoted as *mp*_*i*_.
*gt*	Real position of unmanned vehicle *a*_*j*_, denoted as *gt*_*j*_
*pp*	The ground-truth during training and the expected position during task execution
*puwb*	UWB position of unmanned vehicle *a*_*j*_, noted as *puwb*_*j*_
*pcamera*1	Camera position (IDS ranging) of unmanned vehicle *a*_*j*_, noted as *pcamera*1_*j*_
*pcamera*2	Camera position (Camera ranging) of unmanned vehicle *a*_*j*_, noted as *pcamera*2_*j*_
*pcamera*3	Camera position (Camera and IDS fusion ranging) of unmanned vehicle *a*_*j*_, noted as *pcamera*3_*j*_
*p*	Final position of unmanned vehicle *a*_*j*_ by multi-sensor fusion, noted as *p*_*j*_
*p*_*lst*	The bit pose stored by unmanned vehicle *a*_*j*_ in ascending time order, a list, noted as *p*_*lst*_*j*_
*p*_*v*	The id of the previous of unmanned vehicle *a*_*j*_, noted as *p*_*v*_*j*_
*a*_*v*	The id of the after of unmanned vehicle, noted as *a*_*v*_*j*_
*x*,*y*,*z*,*w*	Thresholds
*r*	The role of unmanned vehicle *a*_*j*_, (leader or follower), noted as *r*_*j*_
*s*	The state of unmanned vehicle *a*_*j*_, (stuck or normal), noted as *s*_*j*_
*fs*	The following method of unmanned vehicle *a*_*j*_ (goal following or trajectory following), noted as *fs*_*j*_
*tp*	The destination location (m) of unmanned vehicle *a*_*j*_, noted as *tp*_*j*_
*tp*_*lst*	The destination location stored by unmanned vehicle *a*_*j*_ in ascending time order, a list, noted as *tp*_*lst*_*j*_
*υ*	The linear velocity (m/s) of unmanned vehicle *a*_*j*_ traveling towards its destination, denoted as *υ*_*j*_
*ω*	The angular velocity (rad/s) of unmanned vehicle *a*_*j*_ traveling towards its destination, denoted as *ω*_*j*_
*d*	The ED (m) of unmanned vehicle *a*_*j*_ from *tp*_*j*_ to *p*_*j*_, denoted as *d*_*j*_
*D*_1_	The ranging of unmanned vehicle *a*_*j*_ of *p*_*v*_*j*_ by IDS (m), denoted as $D_{1_{j}}$
*D*_2_	The ranging of unmanned vehicle *a*_*j*_ of *p*_*v*_*j*_ by Camera (m), denoted as $D_{2_{j}}$
*w*_*uwb*_	Confidence degree of UWB position
*w*_*camera*1_	Confidence degree of Camera position (IDS ranging)
*w*_*camera*2_	Confidence degree of Camera position (Camera ranging)
*w*_*camera*3_	Confidence degree of Camera position (Camera and IDS fusion ranging)
*f*_*uwb*_	Filters whether UWB position is available for fusion, (0 or 1).
*f*_*camera*1_	Filters whether Camera position (IDS ranging) is available for fusion, (0 or 1).
*f*_*camera*2_	Filters whether Camera position (Camera ranging) is available for fusion, (0 or 1).
*f*_*camera*3_	Filters whether Camera position (Camera and IDS fusion ranging) is available for fusion, (0 or 1).

#### Experiment scenario

**Fig 3:**
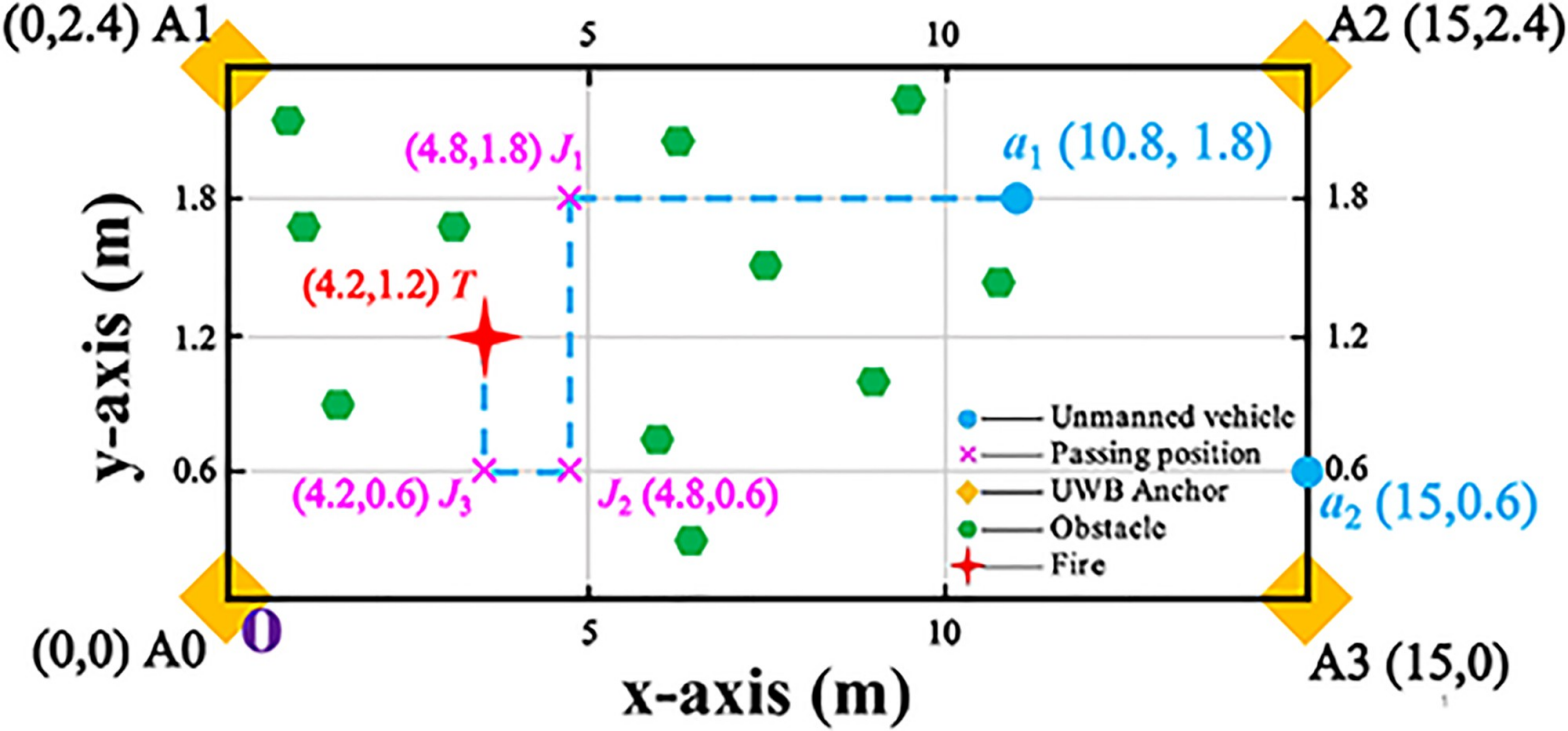
Experiment scenario. The size of this experiment scenario is 15m × 2.4m. Multiple obstacles are also placed within this experiment scenario.

This experimental sets the experimental scenario as a rectangular environment, as shown in Fig 3. This scenario is a rectangular base station laid by four UWB anchors located at the same horizontal position. One of anchors is used as the origin of rectangular.

#### Experiment platform and equipment

The experiments in this paper are conducted By taking the Chengdu Kestrel Artificial Intelligence Institute’s Interface Description Language(IDL)-Mapping tool (KIS-CORBA) as a cross-language data communication protocol, acquiring real-time information with multiple sensors, and publishing or subscribing to the information through the serial port of the Robot Operating System (ROS) to finally implement GBBopen to control firefighting equipment. This IDL is the IDL of Object Management Group (OMG).

**1) Experiment Platform**. All experiments require a PC platform and an ARM-embedded platform. The ARM-embedded platform used in this experiment is the Raspberry Pi (RPI). The Python environment (3.7.0), KIS-CORBA, and ROS platforms are installed into the RPI. The Visual Studio environment (VS2017), KIS-CORBA, Lisp environment (Allegro Common Lisp 10.1 express), LinkTrack technology (NoopLoop for NAssistant applications), and GBBopen are installed on the PC.

**Fig 4:**
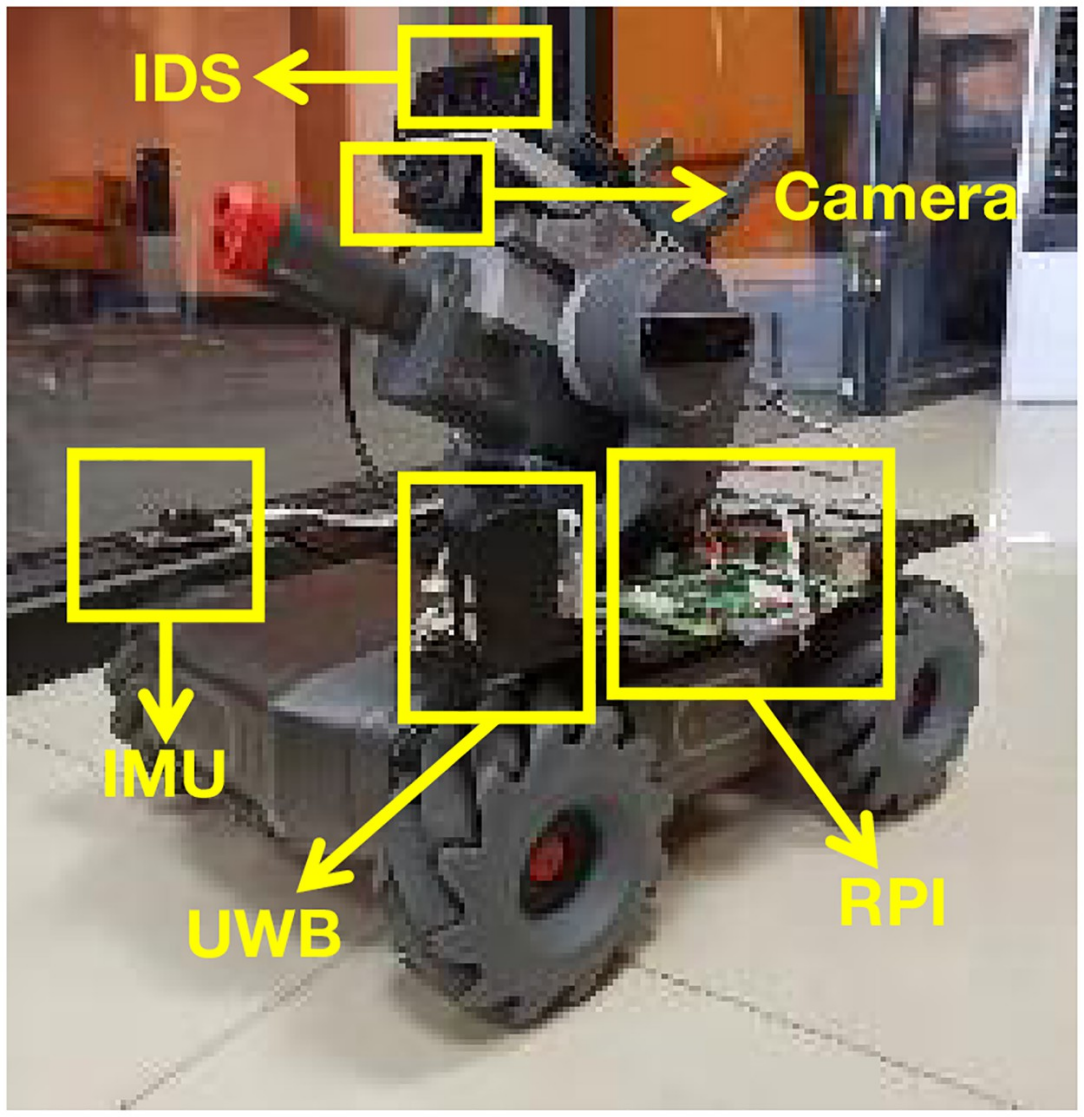
Firefighting equipment. An unmanned vehicle (RoboMaster).

**2) Experiment Equipment**. This experiment uses an unmanned vehicle (RoboMaster) as the firefighting equipment for the FERD system, as shown in Fig 4.

**i. Sensors**. Each piece of unmanned vehicle carried five items, including an IDS, a camera, an UWB anchor, a module of an IMU, and a RPI, as shown in Fig 4.

**ii. Application Programming Interface (API)**. DJI-Innovations (DJI) provides several API instances (https://robomaster-dev.readthedocs.io/zh_CN/latest/) written in Python. This experiment is conducted to realize communication between the unmanned vehicle and GBBopen and to control the wheels of the unmanned vehicle. Because unmanned vehicle is written in Python and GBBopen is written in Lisp, cross-language communication is required. These experiments focused on the communication between Python and Lisp and finally applied the FERD system.

**iii. Attributes**. Various attributes exist for each firefighting task and each unmanned vehicle. These attributes can be expressed as follows:

$$m_{i}=\left\{num,mp\right\}$$ (12)

$$a_{i}=\{id,gt,p,p\_lst,puwb,p\_v,pcamera1,pcamera2,pcamera3,a\_v,r,s,tp,\upsilon ,\omega ,d,fs,D_{1},D_{2}\}$$ (13)

**iv. Data transmission**. The remote server is connected to control the unmanned vehicle through Secure Shell (SSH). The position of the unmanned vehicle, the three-axis attitude angle, and the distance from the front are obtained through UWB, IMU, and IDS, respectively. Among them, UWB achieves high-precision indoor positioning by sending ultra-short pulse signals that exploit multipath propagation and the Doppler effect in indoor environments. The unmanned vehicle obtains images through the camera’s API and embeds a coordinate transformation program to output coordinates. The ROS serial port then reads the obtained data and publishes it in the form of topics. Finally, they are transmitted to GBBopen through KIS-CORBA. The data of the unmanned vehicle is sent to GBBopen, including pose, velocity, distance, etc. GBBopen sends the data to the unmanned vehicle, such as target point, speed, etc.

**v. Pose information**. Positioning errors occur due to various factors (e.g., the deployment mode of the base station, obstracles and the weakening of the signal) in the indoor environment. Therefore, the experiment assumes that the information measured by the UWB and IMU is the actual location information of the unmanned vehicle. In Fig 3, the number of unmanned vehicles is 2. The position of two unmanned vehicles are (10.8, 1.8) and (15,0.6), respectively. And the position of the firefighting task is (4.2, 0.6).

#### Experiment contents

In FERD system, the fusion norms are designed, which guide each unmanned vehicle to perform tasks and the fusion positioning of multiple sensors. Briefly introduce the 22 norms that already exist within FERD system. Norm *r*_1_ creates multiple instances of the unmanned vehicle. Norm *r*_2_ and *r*_3_ are rules for setting the switching ways and sorting the fire locations. The switching ways include Command Mode and Automatic Mode. Norm *r*_4_ ∼ *r*_8_ are rules about the following strategy. Among them, Norm *r*_4_ and *r*_5_ are the ways to determine the following of the unmanned vehicle *a*_*i*_. Norms *r*_7_ and *r*_8_ are parameters to set the unmanned vehicle *a*_*i*_ following mode. Norm *r*_9_ ∼ *r*_13_ are rules about the Mobilization Strategy. These strategies include Formation, Departure, and Return To the Team. Norm *r*_12_ and *r*_13_ are rules for setting the role of the unmanned vehicle *a*_*i*_. Norm *r*_14_ ∼ *r*_16_ are three rules about the Motion Strategy. Here it is recommended that the linear velocity of the unmanned vehicle is between 0.05 ∼ 0.5m/s. The angular velocity of the unmanned vehicle is between 0 ∼ 90rad/s. Norm *r*_17_ ∼ *r*_19_ are the three rules of the Obstacle Avoidance Strategy. Norm *r*_20_ ∼ *r*_22_ are rules about Evolutionary Strategy. Table 4 shows the added norms and revised norms within FERD system in this paper. Norm *r*_14_ ∼ *r*_16_ are rules about three revisions of the Motion Strategy. Norm *r*_23_ ∼ *r*_30_ are rules about the Following Strategy. Norm *r*_31_ ∼ *r*_38_ are rules about updating confidence degree and filtering of observations. Norm *r*_39_ ∼ *r*_40_ are rules about different algorithms about the fusion of multiple sensors. For example, the fusion algorithm of the Kalman Filter based confidence degree and the weighted average-based fusion algorithm are applied in this paper. Norm *r*_41_ is the rule for fusion decision-making. This norm is that the fusion results from different algorithms are compared with *gt*_*j*_. The optimal fusion result is selected.

**Table 4:** Norms of fusion moudle.

Norm	Trigger	Action	Expectation
*r*_14_	*d*_*i*_ > *x*	*a*_*i*_ forward	|*puwb*_*i*_ − *pp*| ≤ *y*
*r*_15_	*d*_*i*_ ≤ *x*	*a*_*i*_ stop	|*puwb*_*i*_ − *pp*| ≤ *y*
*r*_16_	*a*_*i*_ and *a*_*j*_ collide	*a*_*i*_ fallback	|*puwb*_*i*_ − *pp*| ≤ *y*
*r*_23_	*D*_1_ > *w*	Set goal following	*fs*_*i*_ = goal following
*r*_24_	*D*_2_ > *w*	Set goal following	*fs*_*i*_ = goal following
*r*_25_	*puwb*_*i*_ = *ϕ* and $pcamera1_{p\_v_{i}}\neq \phi$	Set goal following	*fs*_*i*_ = goal following
*r*_26_	*puwb*_*i*_ = *ϕ* and $pcamera2_{p\_v_{i}}\neq \phi$	Set goal following	*fs*_*i*_ = goal following
*r*_27_	*puwb*_*i*_ = *ϕ* and $pcamera3_{p\_v_{i}}\neq \phi$	Set goal following	*fs*_*i*_ = goal following
*r*_28_	*w*_*uwb*_ ≤ *z* and $pcamera1_{p\_v_{i}}\neq \phi$	Set goal following	*fs*_*i*_ = goal following
*r*_29_	*w*_*uwb*_ ≤ *z* and $pcamera2_{p\_v_{i}}\neq \phi$	Set goal following	*fs*_*i*_ = goal following
*r*_30_	*w*_*uwb*_ ≤ *z* and $pcamera3_{p\_v_{i}}\neq \phi$	Set goal following	*fs*_*i*_ = goal following
*r*_31_	|*puwb*_*i*_ − *pp*| ≤ *y*	update *y*, *w*_*puwb*_, *f*_*puwb*_	Update completed
*r*_32_	|*puwb*_*i*_ − *pp*| > *y*	update *y*, *w*_*puwb*_, *f*_*puwb*_	Update completed
*r*_33_	$|pcamera1_{p\_v_{i}}-pp|\leq y$	update *y*, *w*_*pcamera*1_, *f*_*pcamera*1_	Update completed
*r*_34_	$|pcamera1_{p\_v_{i}}-pp|>y$	update *y*, *w*_*pcamera*1_, *f*_*pcamera*1_	Update completed
*r*_35_	$|pcamera1_{p\_v_{i}}-pp|\leq y$	update *y*, *w*_*pcamera*2_, *f*_*pcamera*2_	Update completed
*r*_36_	$|pcamera1_{p\_v_{i}}-pp|>y$	update *y*, *w*_*pcamera*2_, *f*_*pcamera*2_	Update completed
*r*_37_	$|pcamera1_{p\_v_{i}}-pp|\leq y$	update *y*, *w*_*pcamera*3_, *f*_*pcamera*3_	Update completed
*r*_38_	$|pcamera1_{p\_v_{i}}-pp|>y$	update *y*, *w*_*pcamera*3_, *f*_*pcamera*3_	Update completed
*r*_39_	∃*f*_*puwb*_, *f*_*pcamera*1_, *f*_*pcamera*2_, *f*_*pcamera*3_ ≠ *ϕ*	Confidence degree based Kalman filter fusion	Fusion completed
*r*_40_	∃*f*_*puwb*_, *f*_*pcamera*1_, *f*_*pcamera*2_, *f*_*pcamera*3_ ≠ *ϕ*	Based on weighted average fusion	Fusion completed
*r*_41_	Fusion completed	Fusion Decision	*p*_*j*_ ≠ *ϕ*

In indoor scenarios with multiple obstacles, each unmanned vehicle chooses the appropriate norms based on its state and assists in firefighting tasks through the sensors it carries itself. This experiment sets a fixed route for two unmanned vehicles. They pass through (4.8,1.8), (4.8,0.6), and (4.2,0.6) in turn. Eventually reaching the fire location (4.2, 1.2), they perform the fire extinguishing task.

#### Experiment limitations

The experiment design can verify the effectiveness of the proposed module, but this module has certain limitations (e.g., timeliness). In this experiment, the relative velocity between two unmanned vehicles is set to check the real-time performance. Specifically, the relative velocity of two unmanned vehicles is used as an independent variable. The fusion results of multiple sensors are used as the dependent variable. Finally, the fusion results of multiple sensors are compared with the real values. This experiment sets different relative velocities between two unmanned vehicles to perform the fire extinguishing task. The relative velocities in this experiment are 0.03m/s, 0.06m/s, 0.09m/s, 0.12m/s, 0.15m/s, and 0.2m/s, respectively.

#### Evaluation metrics

In this experiment, the FER is proposed as the fusion index of this fusion module. GBBopen receives the number of localization coordinates of all sensors simultaneously, expressed by Z. Among them, the distance between the coordinates localized by UWB and the ground truth is large; while the distance between the coordinates localized by other sensors and the ground truth is small. The distance between the coordinates localized by other sensors and the ground truth is small. The number of coordinates of UWB localization is larger than the distance to the ground truth, denoted by z. The FER is expressed as $\frac{Z-z}{Z}$ × 100%. With effectively fused data, one evaluation index is the error range of ED and MSE between observed values and the ground truth of a single sensor. Another evaluation index is the error range of ED and MSE between the fusion results of multiple sensors and their ground truth.

### Steps

First, this experiment analyzes the different sensors in different scenarios. Next, the important parameters inside the norms are trained in indoor scenario with multiple obstacles. Finally, the two unmanned vehicles follow a set fixed route and assist in firefighting tasks.

#### Observed values of different sensors in different scenarios

This step analyzes the different sensors in different scenarios. And the observed values are compared with the real values.

**1) UWB Positioning**. A unmanned vehicle *a*_1_ carries a UWB anchor to position itself. *a*_1_ starts with the real position (7.8,1.8). *a*_1_ is moved 10 times from right to left at 600mm intervals in sequence. *a*_1_ ends at the real position (2.4,1.82). The real positions of *a*_1_ are (7.8,1.8), (7.2,1.8), (6.6,1.8), (6,1.8), (5.4,1.8), (5.4,1.8) (4.8,1.8), (4.2,1.8), (3.6,1.8), (3,1.78), (2.4,1.82). Finally, the values of the positioning are compared with the real values.

**2) IDS and Camera Ranging**. First, the mapping relationship between world coordinates and pixel coordinates is obtained by Formula 6. The internal and external reference matrices of the camera are obtained by camera calibration. Then, *a*_1_ carries Camera and IDS to range unmanned vehicle *a*_2_. *a*_2_ moves 10 times in sequence at 600mm intervals from 600∼6000mm. The distances between *a*_1_ and *a*_2_ are 600mm, 1200mm, 1800 mm, 2400mm, 3000mm, 3600mm, 4200mm, 4800mm, 5400mm, and 6000mm respectively. Finally, the values of the ranging are compared with the real values.

**3) UWB, IDS and Camera Positioning**. *a*_1_ carries Camera and IDS to range *a*_2_. Then, two ranging results are fused. Next, *a*_1_ was positioned using both UWB and Camera. *a*_2_ robot positioning starts with the real position (7.8,1.8). *a*_2_ is moved 10 times from right to left at 600mm intervals in sequence. *a*_2_ ends at the real position (2.4,1.82). The real positions of *a*_2_ are (7.8,1.8), (7.2,1.8), (6.6,1.8), (6,1.8), (5.4,1.8), (5.4,1.8) (4.8,1.8), (4.2,1.8), (3.6,1.8), (3,1.78), (2.4,1.82). Finally, the values of the positioning are compared with the real values.

#### Train error thresholds and confidence degrees

The accuracy and error of the observed values of each sensor are different. Each sensor also contributes differently to the fusion of multiple sensors. Therefore, some parameters of some norms are trained. They include the confidence degree of each sensor and the error threshold of all sensors. In this experiment, the number of training is 10. In the initial stage of training, the confidence degree of each sensor is equal. And the error threshold of all sensors is set to null. The results at the end of the first training are the initial values for the second training. Until the end of the tenth training session, the trained results are the initial values of the unmanned vehicles performing tasks.

#### Performing tasks

The two unmanned vehicles follow a set fixed route and assist in firefighting tasks through the parameters of the norms that have been trained and the sensors that each carries.

### Results and analysis

The results of the experiment steps are given in this paper. Then, the experiment results are analyzed.

#### Observed values of different sensors in different scenarios

The results of the observed values and true values are analyzed by different perspectives (quantitative and qualitative) in different scenarios.

**Fig 5:**
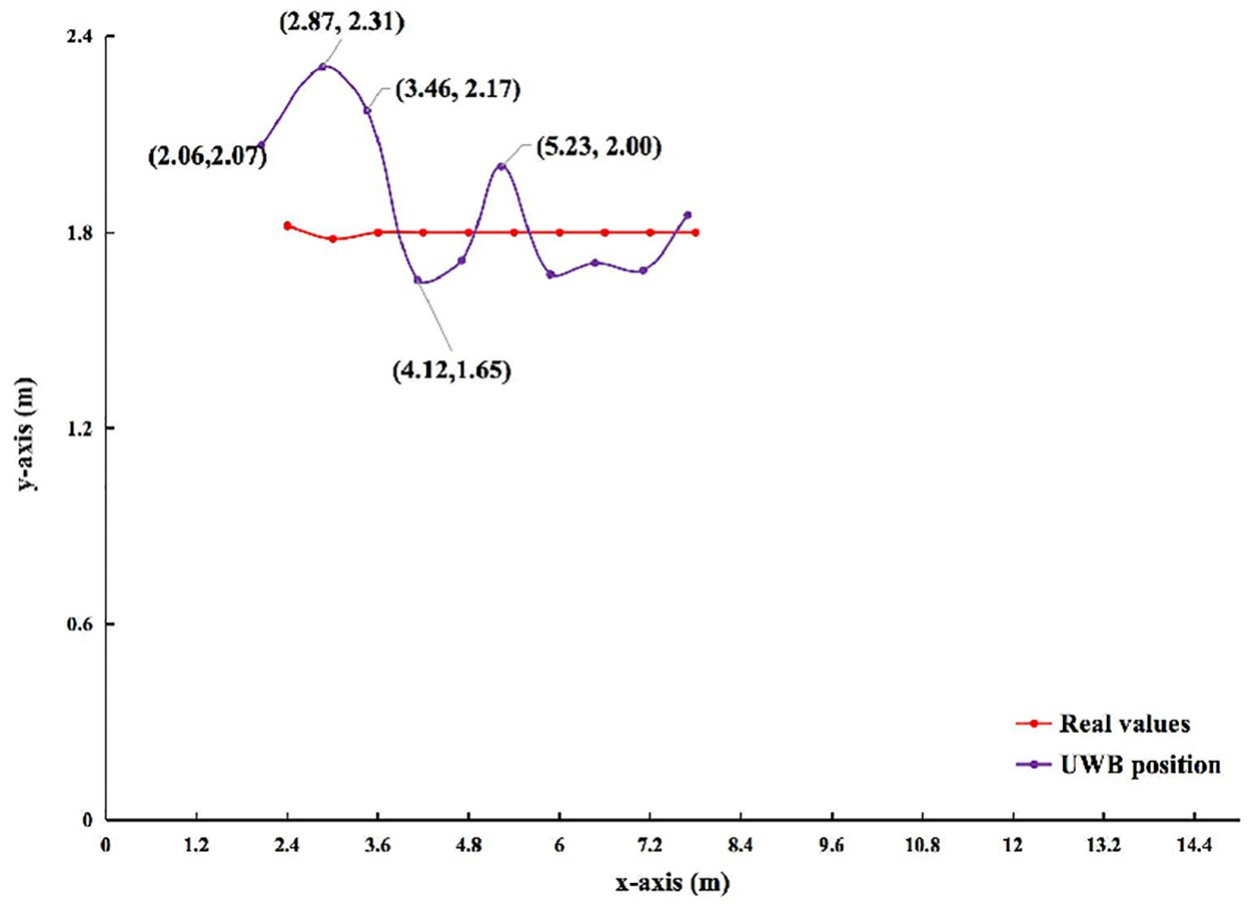
In indoor scenario without no obstacle. Comparison of UWB position and real values in different scenarios.

**Fig 6:**
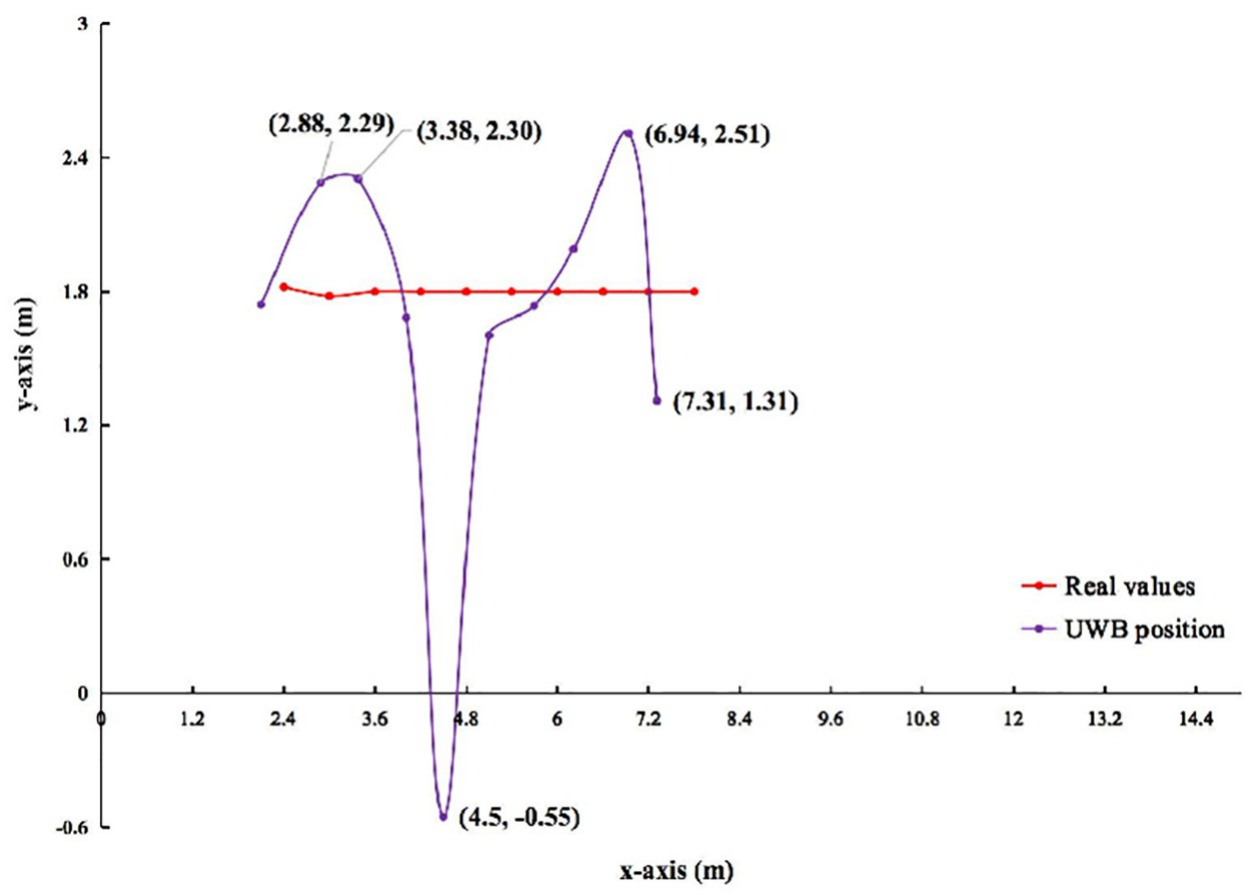
In indoor scenario with multiple obstacles. Comparison of UWB position and real values in different scenarios.

**1) UWB Positioning**. Figs 5 and 6 show the results of the observed values and real values of unmanned vehicle *a*_1_ analyzed by qualitative. Horizontal axis indicates the length of the rectangle. Vertical axis indicates the width of the rectangle. The positioning of *a*_1_ are compared 10 times in order from right to left. The red line indicates the real values of *a*_1_. The purple line indicates the observed value of UWB. Table 5 shows the results of the observed values and true values of unmanned vehicle *a*_1_ analyzed by quantification. Vertical indicates the number of experiments. Horizontal indicates the data analysis from different scenarios.

**Table 5:** Data analysis of between observed values of UWB position and real values.

Experiment	In indoor scene without obstacles							In indoor scene with multiple obstacles						
	x-axis			y-axis			ED	x-axis			y-axis			ED
	Error (m)	SD	MSE (*m*^2^)	Error (m)	SD	MSE (*m*^2^)	Error (m)	Error (m)	SD	MSE (*m*^2^)	Error (m)	SD	MSE (*m*^2^)	Error (m)
1	-0.11	0.01	**0.01**	0.05	0.07	**0.01**	**0.01**	**-0.49**	0.04	**0.24**	-0.49	0.35	0.36	0.48
2	-0.10	0.01	**0.01**	-0.12	0.04	0.02	0.02	-0.26	0.02	0.07	**0.71**	0.13	0.51	0.57
3	-0.13	**0.00**	0.02	-0.10	**0.03**	**0.01**	0.03	-0.39	**0.13**	0.17	0.19	0.29	0.12	0.19
4	-0.12	**0.00**	**0.01**	-0.13	**0.03**	0.02	0.03	-0.31	0.09	0.10	-0.07	0.35	0.13	0.10
5	-0.17	0.01	0.03	0.20	**0.10**	0.05	0.07	-0.30	0.02	0.09	-0.20	0.16	0.07	0.13
6	-0.10	0.01	**0.01**	-0.09	0.05	**0.01**	0.02	-0.30	0.12	0.11	**-2.35**	**0.90**	**6.34**	**5.62**
7	**-0.08**	0.01	**0.01**	**-0.15**	0.05	0.02	0.03	-0.18	0.01	0.03	-0.12	0.10	**0.02**	**0.05**
8	-0.14	0.02	0.02	0.37	**0.10**	0.15	0.16	-0.22	0.06	0.05	0.50	0.23	0.31	0.30
9	-0.13	**0.03**	0.02	**0.53**	0.08	**0.28**	**0.29**	**-0.12**	**0.01**	**0.01**	0.51	**0.06**	0.26	0.27
10	**-0.34**	0.01	**0.12**	0.25	0.06	0.06	0.18	-0.30	0.11	0.10	-0.08	0.23	0.06	0.10

In Fig 5, the red line is smoother, while the purple line has fluctuations. So, there is instability in the observed values of UWB. In Fig 6, the red line is smoother. the purple line fluctuates more. So, there is instability in the observed values of UWB. And it is less stable than in an indoor scenario without obstacles. In Table 5, the quantitative results are analyzed as follows. In an indoor scenario without obstacles, the error range on the x-axis, y-axis, and European distance is -0.34∼-0.08 m, -0.15∼0.53 m, 0.01∼0.29 m. The SD on the x-axis and y-axis ranges from 0.0∼0.03 m, and 0.03∼0.10 m, respectively. The range of MSE on the x-axis and y-axis is 0.01∼0.12*m*^2^ and 0.01∼0.28*m*^2^, respectively. In an indoor scenario with multiple obstacles, the error range on the x-axis, y-axis, and European distance is -0.49∼-0.12 m, -2.35∼0.71 m, 0.05∼5.62 m. The SD on the x-axis and y-axis ranges from 0.01∼0.13 m, and 0.06∼0.90 m, respectively. The range of MSE on the x-axis and y-axis is 0.01∼0.24*m*^2^ and 0.02∼6.34*m*^2^, respectively. This quantitative result illustrates that the observed values of UWB fluctuate more on the y-axis than on the x-axis. The UWB observations fluctuate more in an indoor scenario with multiple obstacles than without obstacles. This is due to the fact that the accuracy of the short edge (y-axis) is worse than the long edge (x-axis) in the unobstructed case. The impact on the ranging accuracy in an indoor scenario with multiple obstacles and the impact of the ranging algorithm can cause the short edge to be affected more than the long edge.

**Fig 7:**
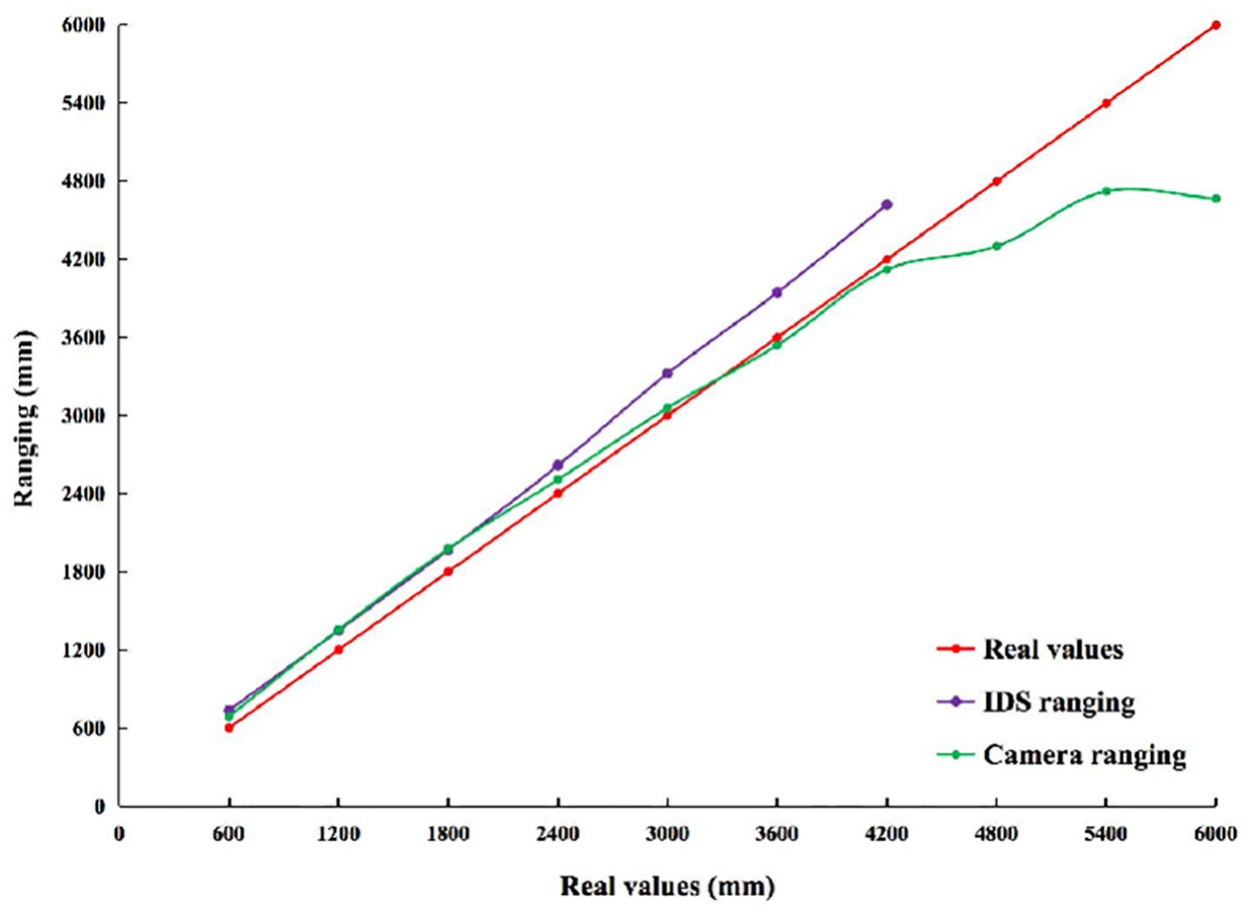
In indoor scenario without no obstacle. Comparison of observed values and real values of ranging by different sensors in different scenarios.

**Fig 8:**
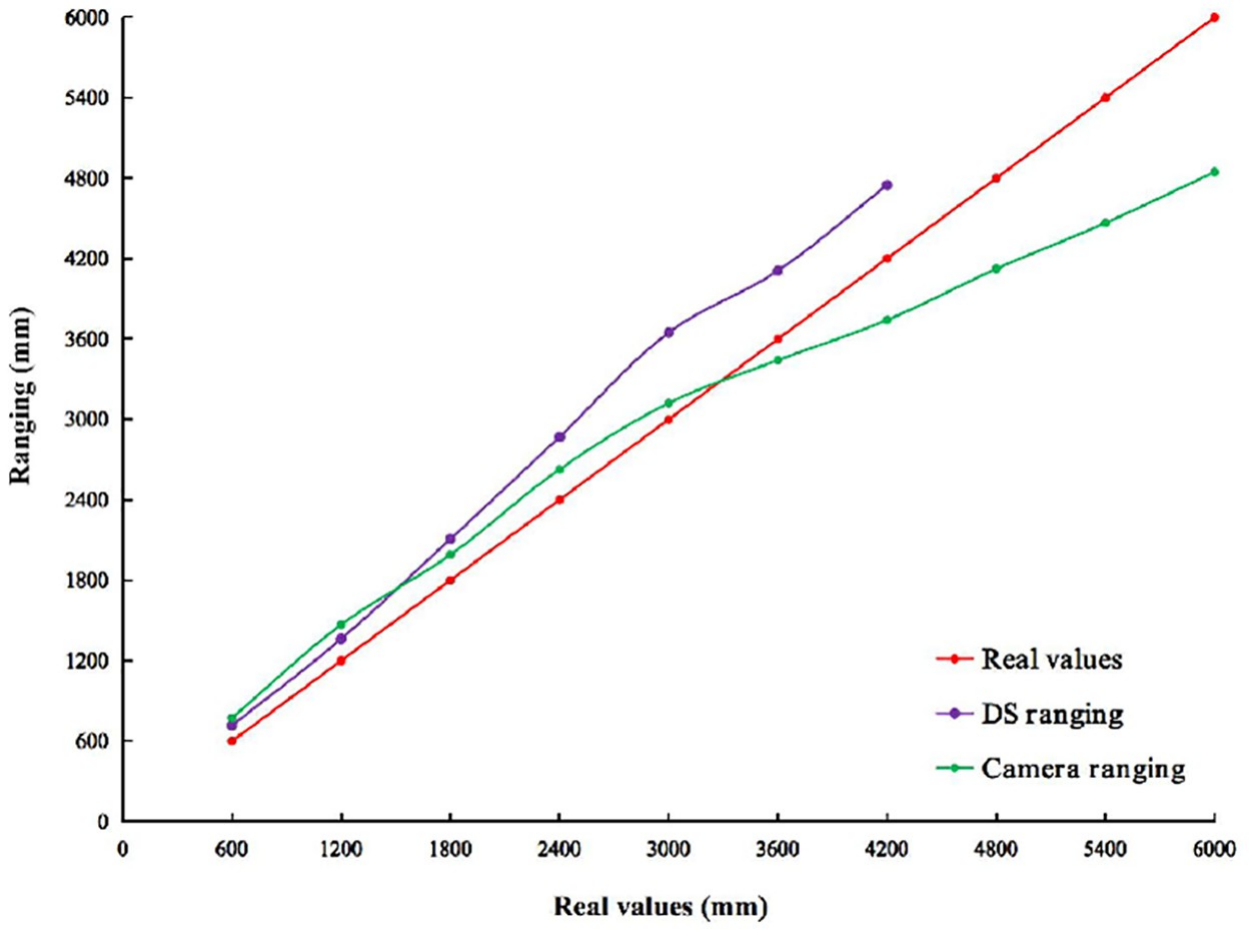
In indoor scenario with multiple obstacles. Comparison of observed values and real values of ranging by different sensors in different scenarios.

**2) IDS and Camera Ranging**. Figs 7 and 8 show the results of the observed values and real values analyzed by qualitative. The real values are distance between *a*_1_ and *a*_2_. The observed values are *a*_1_ ranging *a*_2_ through the carried IDS and the Camera. Horizontal axis indicates the number of experiments. Vertical axis indicates the observed values of ranging. The red line indicates real values. The purple line indicates that observed values are *a*_1_ ranging *a*_2_ through the carried IDS. The green line indicates that observed values are *a*_1_ ranging *a*_2_ through the carried the Camera. Table 6 shows the results of the observed values and real values analyzed by quantification. Vertical indicates the real values. Horizontal indicates the error between observed values and real values by different sensors in different scenarios.

**Table 6:** Data analysis of ranging in different sensors.

Real values	Error in an indoor scene without no obstacle		Error in an indoor scene with multiple obstacles	
	IDS and Real values (mm)	Camera and Real values (mm)	IDS and Real values (mm)	Camera and Real values (mm)
600	**134.86**	87.88	**115.74**	173.11
1200	148.60	156.01	162.56	**268.55**
1800	168.26	176.95	307.80	190.15
2400	216.77	107.84	469.80	226.80
3000	323.51	59.20	**647.74**	120.72
3600	345.50	-60.42	509.71	-158.64
4200	**420.35**	-79.56	547.93	-458.22
4800	No data	**489.13**	No data	-677.16
5400	No data	-678.48	No data	-934.05
6000	No data	**-1334.71**	No data	**-1154.43**
SD	111.70	489.13	202.34	529.73

In Figs 7 and 8, there is no data for ranging by IDS in 4800∼6000mm, while the data is available for ranging by Camera. The experimental results show that ranging by IDS does not work, while it works by Camera in 4800∼6000mm. The experimental results show that observed values of ranging by IDS are smaller than the real values. Table 6 is analyzed from the quantitative perspective as follows. In an indoor scenario without obstacles, the error range between ranging by IDS and real values is 134.86∼420.35mm. The SD of these errors is 111.70. The range of error between ranging by Camera and real values is -1334.713∼489.13mm. The SD of these errors is 489.13. The SD of the Camera is 4.38 times higher than the IDS. In an indoor scenario with multiple obstacles, the error range between ranging by IDS and real values is 115.74∼647.74. The SD of these errors is 202.34. The range of error between ranging Camera and real values is -1154.43∼268.55mm. The SD of these errors is 529.73. The SD of ranging by Camera is 2.62 times higher than ranging by IDS. These results show that ranging by IDS and Camera has almost no effect in different scenarios. However, ranging by IDS is more stable than ranging by Camera. This is due to the detection of the target by a Camera that has errors (i.e., errors in the pixel width), resulting in errors in the numerator of Formula (7).

**Fig 9:**
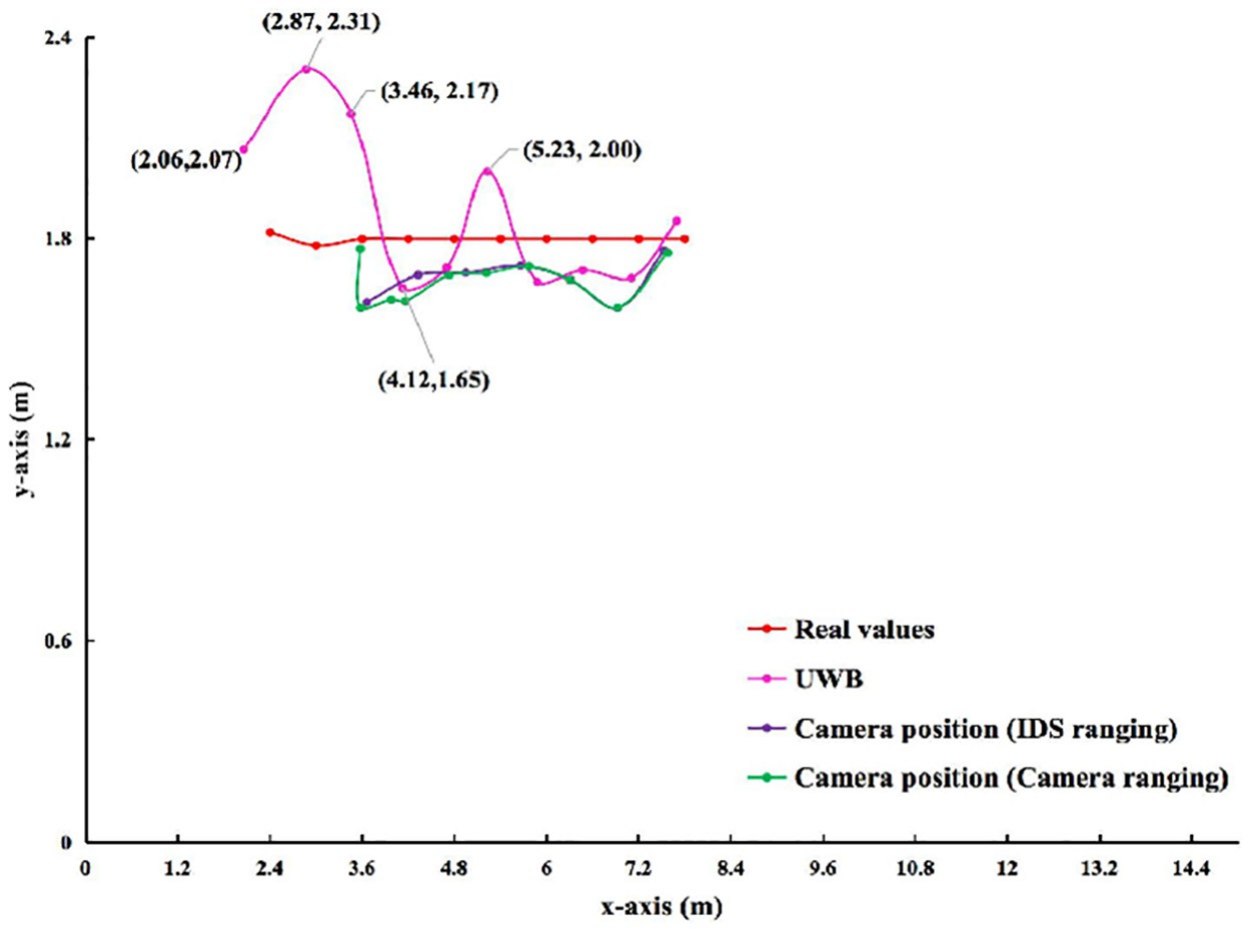
In indoor scenario without no obstacle. Comparison of observed values and real values in different scenarios.

**Fig 10:**
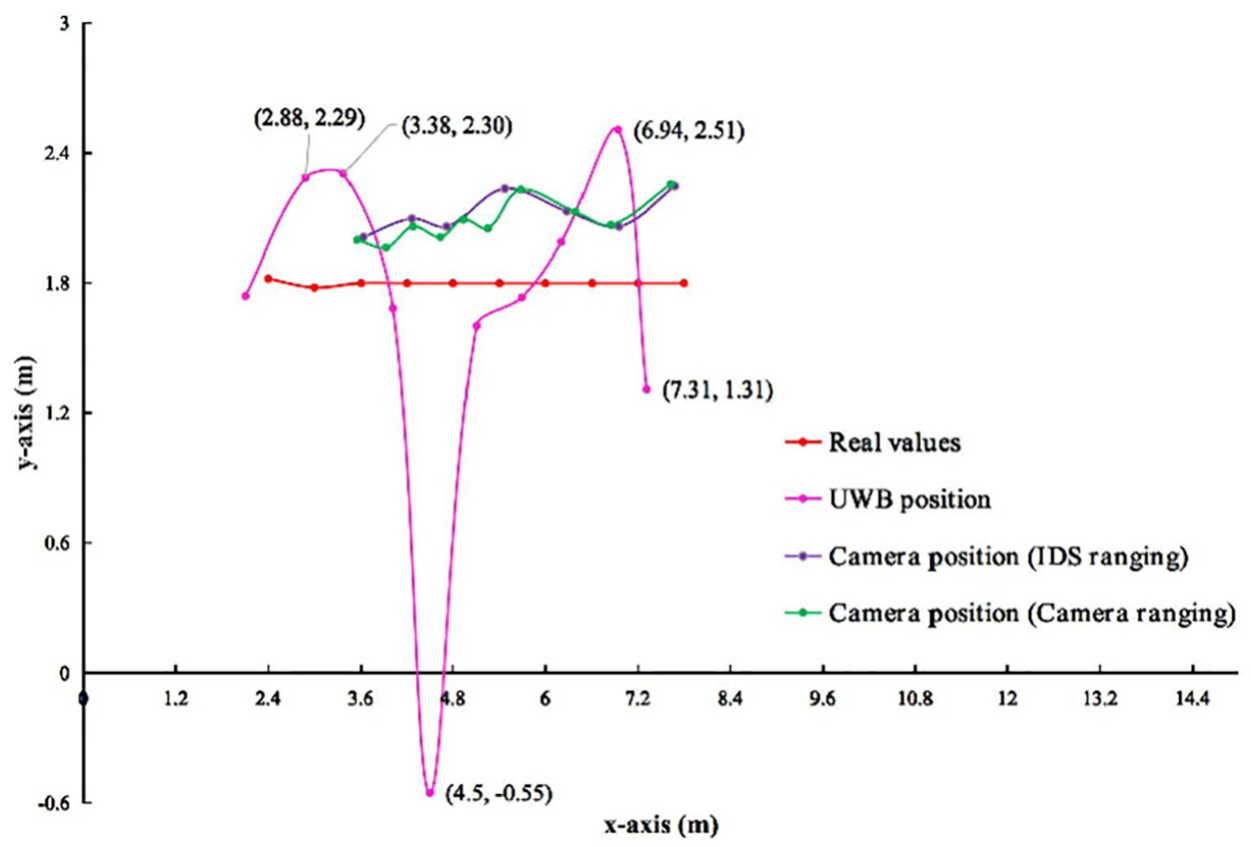
In indoor scenario with multiple obstacles. Comparison of observed values and real values in different scenarios.

**3) UWB, IDS and Camera Positioning**. Figs 9 and 10 show the results of the observed values and real values analyzed by qualitative. Horizontal axis indicates the length of the rectangle. Vertical axis indicates the width of the rectangle. The unmanned vehicle *a*_2_ is compared ten times in order from right to left. The red line indicates the real values of *a*_2_. The pink line indicates the observed values of *a*_1_ by UWB to position itself. The purple line indicates that *a*_1_ by IDS to range *a*_2_. At the same time, the observed values of *a*_1_ by Camera to position *a*_2_. The green line indicates the monocular range results of *a*_1_ by Camera to *a*_2_. Also, the observed values of *a*_1_ by Camera to position *a*_2_.

Tables 7 and 8 shows the data analysis between the observed values and real values by different sensors in different scenarios. In these table, vertical represents real values, horizontal indicates the error between observed values and real values by different sensors.

**Table 7:** Data analysis of observed values and real values by different sensors in indoor scenario without obstacles.

Real value		Error of UWB			Error of Camera position(IDS ranging)			Error of Camera position (Camera ranging)		
x-axis (m)	y-axis (m)	x-axis (m)	y-axis (m)	ED (m)	x-axis (m)	y-axis (m)	ED (m)	x-axis (m)	y-axis (m)	ED (m)
7.8	1.8	-0.11	0.05	**0.01**	-0.30	**-0.03**	**0.07**	-0.22	**-0.04**	0.05
7.2	1.8	-0.10	-0.12	0.02	**-0.27**	**-0.21**	0.12	-0.28	**-0.21**	0.12
6.6	1.8	-0.13	-0.09	0.03	-0.29	-0.12	0.10	**-0.29**	-0.12	0.10
6	1.8	-0.12	-0.13	0.03	-0.34	-0.08	0.12	-0.23	-0.08	0.06
5.4	1.8	-0.17	0.20	0.07	-0.45	-0.10	0.21	-0.18	-0.10	0.04
4.8	1.8	-0.10	-0.09	0.02	-0.47	-0.11	0.23	-0.07	-0.11	**0.02**
4.2	1.8	**-0.08**	**-0.15**	0.03	**-0.54**	-0.19	**0.33**	-0.04	-0.19	0.04
3.6	1.8	-0.14	0.37	0.16	No data	No data	No data	0.38	-0.18	0.18
3	1.78	-0.13	**0.53**	**0.29**	No data	No data	No data	0.58	-0.19	0.37
2.4	1.82	**-0.34**	0.25	0.18	No data	No data	No data	**1.17**	-0.05	**1.38**

**Table 8:** Data analysis of observed values and real values by different sensors in indoor scenario with multiple obstacles.

Real value		Error of UWB			Error of Camera position(IDS ranging)			Error of Camera position (Camera ranging)		
x-axis (m)	y-axis (m)	x-axis (m)	y-axis (m)	ED (m)	x-axis (m)	y-axis (m)	ED (m)	x-axis (m)	y-axis (m)	ED (m)
7.8	1.8	-0.49	-0.49	0.48	-0.12	0.45	0.21	-0.18	0.45	0.24
7.2	1.8	-0.26	0.71	0.57	-0.25	0.26	0.13	-0.36	0.27	0.20
6.6	1.8	-0.39	0.19	0.19	-0.32	0.33	0.21	-0.21	0.33	0.15
6	1.8	-0.31	-0.07	0.10	-0.53	0.43	0.47	-0.32	0.43	0.29
5.4	1.8	-0.30	-0.20	0.13	-0.68	0.26	0.53	-0.15	0.25	0.09
4.8	1.8	-0.30	-2.35	5.62	-0.54	0.30	0.37	0.13	0.29	0.10
4.2	1.8	-0.18	-0.12	0.05	-0.57	0.21	0.37	0.44	0.21	0.23
3.6	1.8	-0.22	0.50	0.30	No data	No data	No data	0.68	0.26	0.53
3	1.78	-0.12	0.51	0.27	No data	No data	No data	0.93	0.18	0.89
2.4	1.82	-0.30	-0.08	0.10	No data	No data	No data	1.15	0.18	1.35

In Figs 9 and 10, the trend of red line is flat, while the trends of pink, purple, and green lines are fluctuation. The trend of pink line are greater than purple and green lines. When the real coordinates of *a*_2_ (8.4,1.8) and *a*_1_ are (3.6,1.8), (3,1.78) and (2.4,1.82) in that order, there is no data for ranging by IDS. So no observed values are Camera position (IDS ranging). While the data is available for ranging by Camera, so observed values are Camera position (Camera ranging). In Tables 7 and 8, this experiment compares the observed values of *a*_1_ by different sensors with the real values. In indoor scenario without obstacles, the error ranges of *a*_1_ by UWB position itself on the x-axis, y-axis, and ED are -0.34∼-0.08 m, -0.15∼0.53 m, and 0.01∼0.29 m, respectively. The range error of *a*_1_ by Camera position (IDS ranging) position *a*_2_ on the x-axis, y-axis, and ED is -0.54∼-0.27 m, -0.21∼-0.03 m, 0.07∼0.33 m, respectively. The range error of *a*_1_ by Camera position (Camera ranging) position *a*_2_ on the x-axis, y-axis, and ED is -0.29∼1.17 m, -0.21∼-0.04 m, 0.02∼1.38 m, respectively. In indoor scenario with multiple obstacles, The range error *a*_1_ by UWB position itself on the x-axis, y-axis, and ED is -0.49∼-0.12 m, -2.35∼0.71 m, 0.05∼5.62 m, respectively. The range error of *a*_1_ by Camera position (IDS ranging) position *a*_2_ on the x-axis, y-axis, and ED is -0.68∼-0.12 m, 0.21∼0.45 m, 0.13∼0.53 m, respectively. The range error of *a*_1_ by Camera position (Camera ranging) position *a*_2_ on the x-axis, y-axis, and ED is -0.36∼1.15 m, 0.18∼0.45 m, 0.09∼1.35 m, respectively.

From the above qualitative and quantitative analysis results, it is clear that the stability of UWB observations varies from scenario to scenario. This experiment allows the IDS and Camera to complement or fuse each other for ranging the unmanned vehicle. When the Camera (IDS ranging) measurement fails, Camera position (Camera ranging) and UWB can complement each other.

#### Train error thresholds and confidence degrees

**Fig 11:**
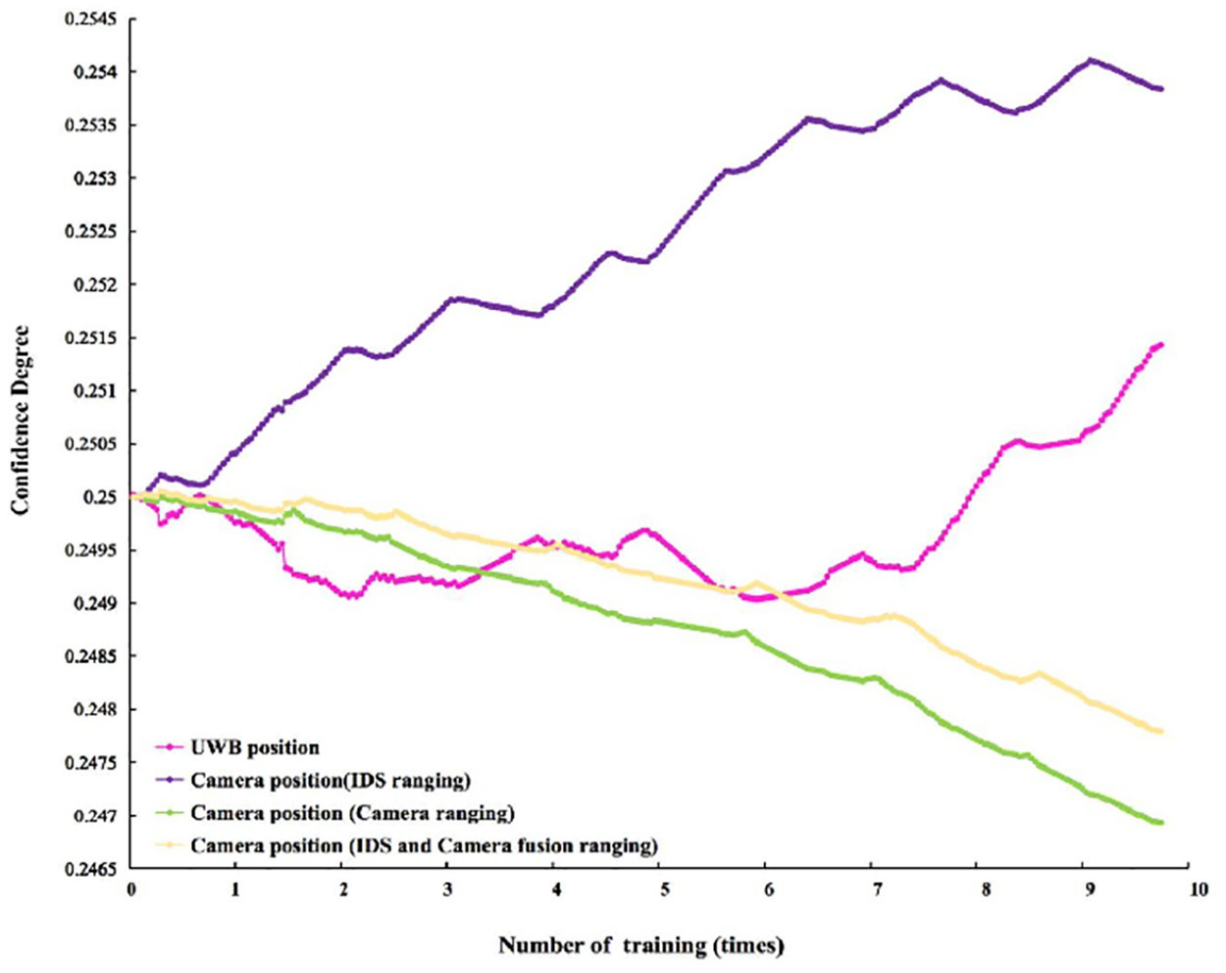
Training confidence. Training variation curves of confidence degree. The different color lines show the change training trend of different sensors’ confidence degrees. The pink line shows the change training trend of UWB positioning. The purple line shows the change training trend of Camera position (IDS ranging). The green line shows the change training trend of Camera position (Camera ranging). The yellow line indicates the change training trend of Camera position (Camera and IDS fusion ranging).

**Fig 12:**
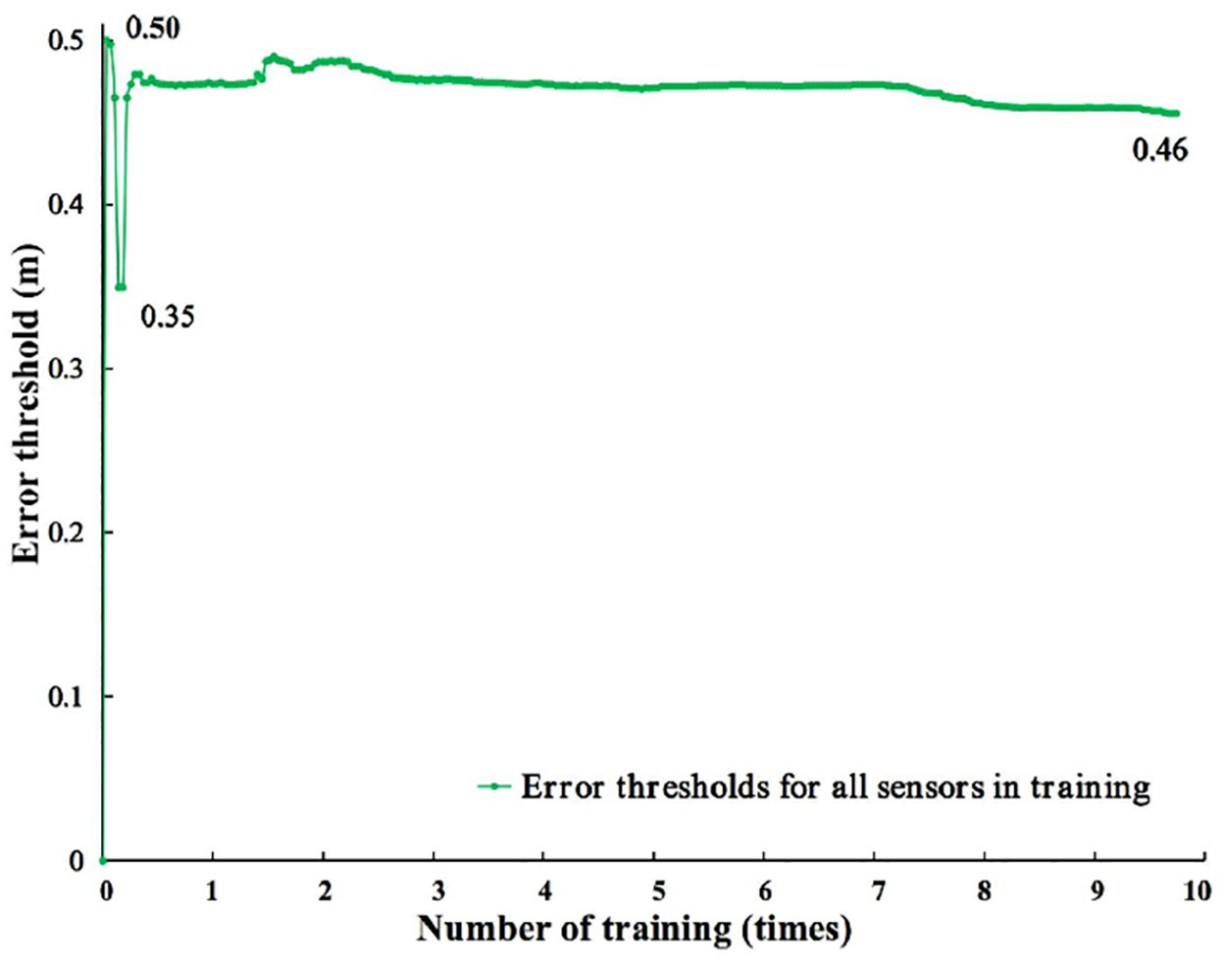
Training error threshold. Training variation curves of error thresholds.

Figs 11 and 12 show the change curve of training confidence degree and error threshold.

In Fig 11, the four different channels have the same confidence degree of 0.25 in the initial stage of training. The confidence degrees of the four different channels gradually differ as the number of training sessions increases. The confidence degree of the Camera position (IDS ranging) shows a fluctuating trend of rapid rise. The confidence degree of UWB’s position shows a fluctuating trend of slow rise. The confidence degree of the Camera position (Camera ranging) shows a slow downward trend. The confidence degree of the Camera position (Camera and IDS fusion ranging) shows a rapid downward trend. After training, their confidence degrees are as follows. Camera position (IDS ranging) has the highest confidence degree of 0.52. This is followed by the confidence degree of UWB position with 0.36. The confidence degree of Camera position (Camera and IDS fusion ranging) was 0.12. The lowest confidence degree of Camera position (Camera ranging) was 0.04. Fig 12 indicates the training trend of the error threshold for all sensors. At the initial stage of training, the error threshold of all sensors is 0 m. In the first training, the training error threshold of all sensors fluctuates greatly. The maximum is 0.50m, and the minimum is 0.35m. With the increase in training times, the fluctuation amplitude of all sensor error thresholds tends to be stable. The stable value reaches 0.47m. After training, the error threshold of all sensors reaches 0.46m.

#### Timeliness

Table 9 shows the timeliness results of the fusion module. The table indicates the relative speeds of the two unmanned vehicles in the horizontal direction. The longitudinal direction indicates whether the position after fusion is lagged and the lag value. It is obvious from the table that there is no lag in the fusion results of multiple sensors when the relative speeds between *a*_1_ and *a*_2_ are set at 0.03m/s, 0.06m/s, 0.09m/s, and 0.12m/s. While the relative speed between *a*_1_ and *a*_2_ is 0.15m/s and 0.2m/s, the fusion results of multiple sensors show lag. At a relative velocity of 0.15m/s, the fusion results differ from the real values by 0.3m. At a relative velocity of 0.2m/s, the fusion results differ from the real values by 2m. From the analysis in the table, it is clear that the relative velocity is within 0.12m/s to ensure the validity of the fusion results of multiple sensors.

**Table 9:** Results of timeliness.

Relative velocity of *a*_1_ and *a*_2_	0.03 (m/s)	0.06 (m/s)	0.09 (m/s)	0.12 (m/s)	0.15 (m/s)	0.2 (m/s)
**Fusion result whether lagging (m)**	No	No	No	No	Yes	Yes
**Lagging value**	—	—	—	—	0.3	2

#### Performing tasks

**Fig 13:**
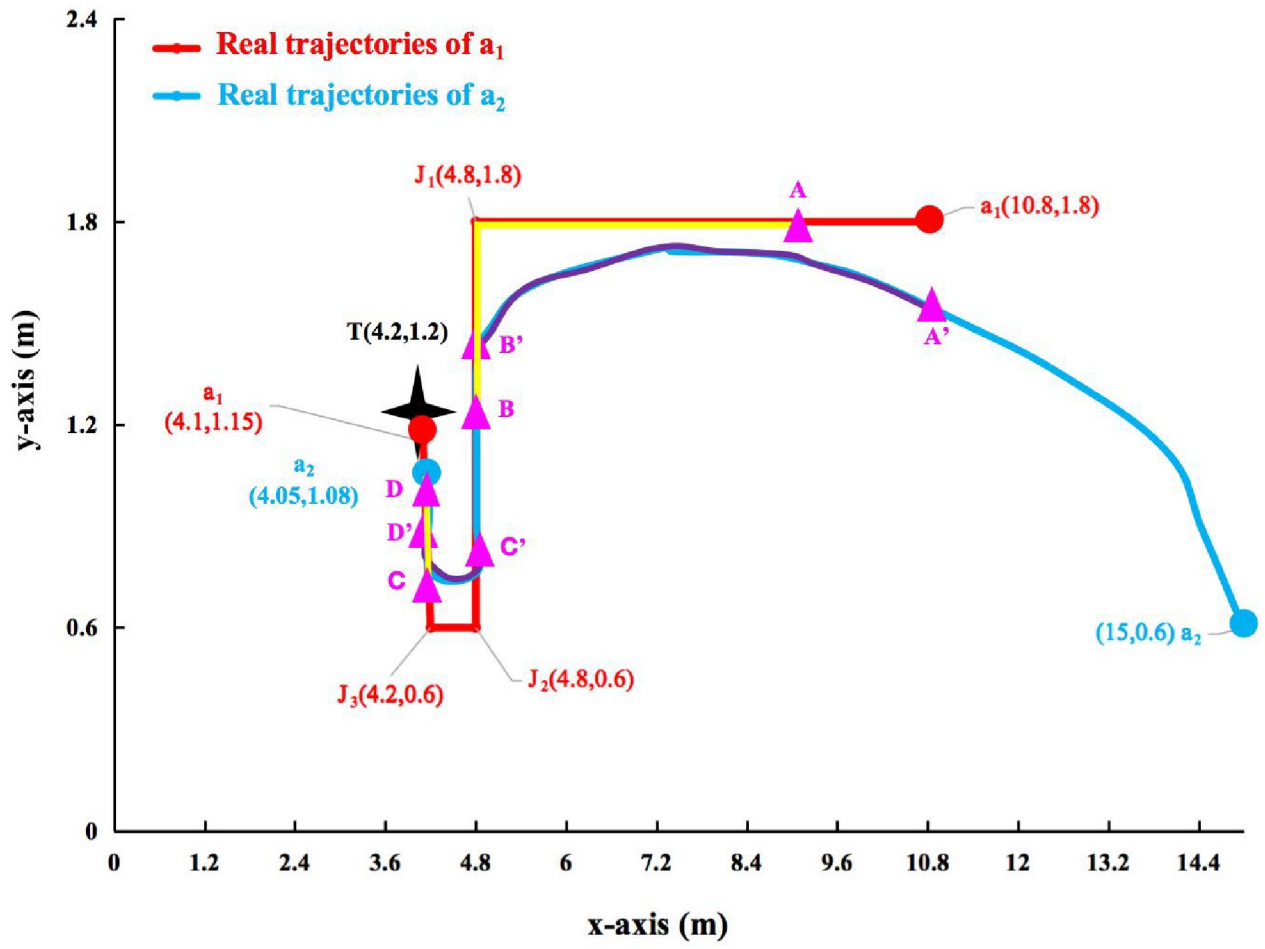
Real trajectory of *a*_1_ and *a*_2_. This figure shows the real trajectory of *a*_1_ and *a*_2_ are perform a task.

**Fig 14:**
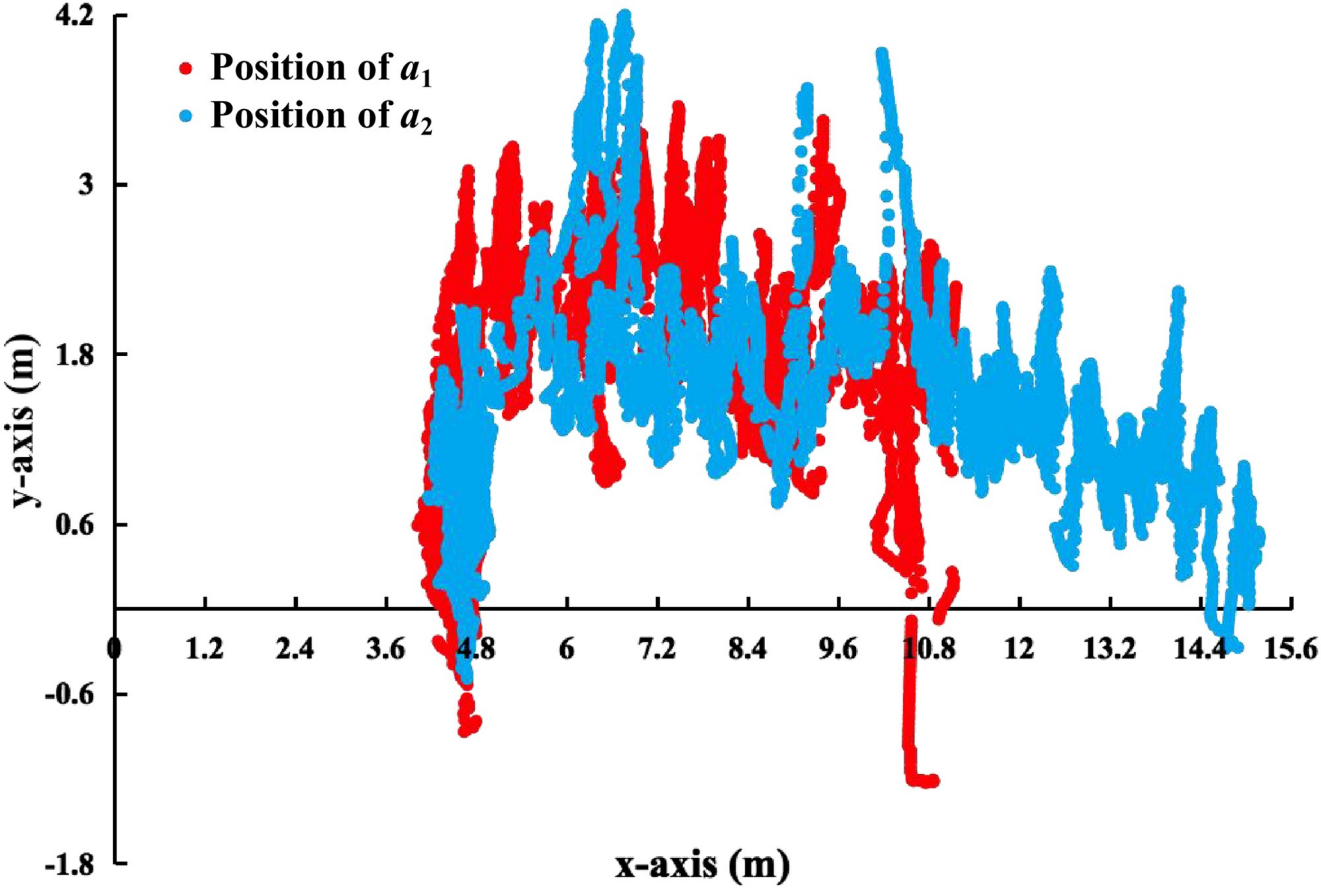
Positioning trajectory of *a*_1_ and *a*_2_ by UWB. The red and blue lines indicate the positioning trajectory of *a*_1_ and *a*_2_, respectively. The red line is available at https://doi.org/10.5061/dryad.31zcrjdrt (DOI: 10.5061/dryad.31zcrjdrt).

**Fig 15:**
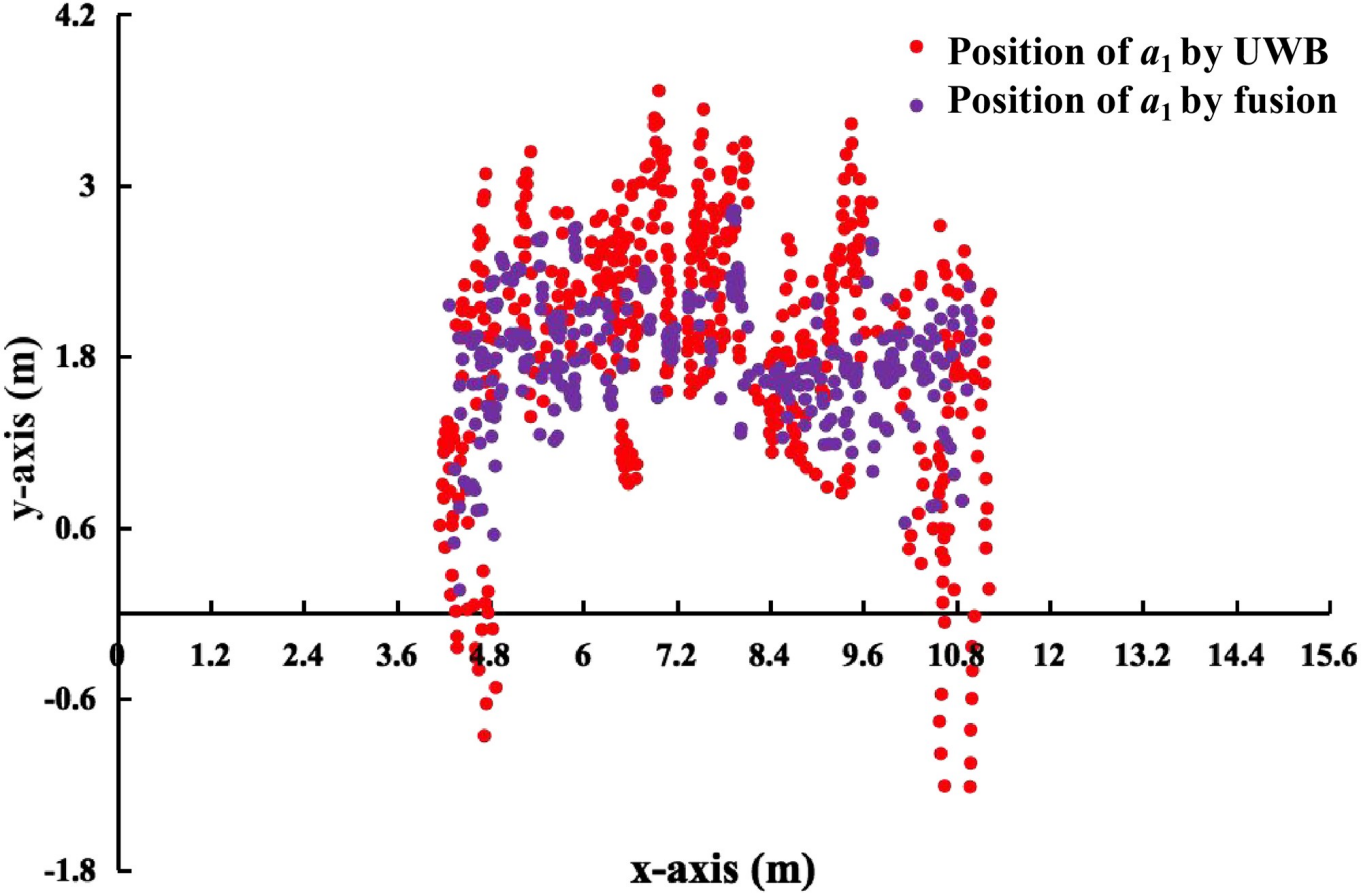
Comparison between UWB positioning and fusion positioning of *a*_1_.

Figs 13–15 shows the visualization results of the FERD system performing task. *a*_1_ and *a*_2_ perform firefighting tasks in an indoor scenario with multiple obstacles. Horizontal axis indicates the length of the rectangle. Vertical axis indicates the width of the rectangle.

In Fig 13, *a*_1_ follow a fixed route. It starts at (10.8,1.8) and passes through *J*_1_, *J*_2_ and *J*_3_ in sequence. Finally, it reaches fire *T* (4.2,1.2) to extinguish the fire. In fact, it is unavoidable that *a*_1_’s wheels have a slight deviation when moving forward. As *a*_1_ moves forward, simultaneously and quickly adjust its deviation, so that *a*_1_ does not differ too much from this route. It is equivalent to *a*_1_ moving along a track. Therefore, the real trajectory of *a*_1_ coincides with the fixed route. The red line indicates the real trajectory of *a*_1_. *a*_2_ starts from (15,0.6). *a*_2_ passes through *A*′, *B*′, *C*′, and *D*′ in that order. *a*_2_ finally reaches (4.05,1.08). The blue line indicates the real trajectory of *a*_2_. The trajectories of *a*_1_ and *a*_2_ are from right to left. The yellow line indicates the *a*_2_ by multiple sensors to observe *a*_1_. The yellow line is also a fusion trajectory fused of *a*_1_.

However, the positioning trajectory obtained by UWB is not consistent with the real trajectory in Figs 13 and 14. This is due to the fact that the experiment is conducted in an indoor environment with many obstacles. The accuracy of UWB positioning is affected by signal interference, reflections, and other factors.

Fig 15 shows the valid coordinates of *a*_1_ localized by different sensors. The figure includes the visualization results of single-sensor and multi-sensor fusion. The red dots are the coordinates of *a*_1_ localized by the UWB carried by *a*_1_. Purple dots are the coordinates localized by the fusion module for *a*_1_. Specifically, *a*_1_ is fused through the positioning coordinates of a single sensor and *a*_2_ through multiple sensors observing the positioning coordinates of *a*_1_. In addition, the Fig 15 is somewhat different from the result of locating *a*_1_ by fusion in Fig 14. The trajectory of *a*_1_ by UWB localization in Fig 14 is a continuous line, as shown in the red line in Fig 14. While it is a discontinuous point, as shown in the red dots in Fig 15. This is due to the fact that the coordinates obtained by GBBopen are different from the frequency of the localization coordinates obtained by UWB. Among them, UWB obtains the positioning coordinates more frequently than the coordinates obtained by GBBopen. And the data obtained by GBBopen is also susceptible to the influence of net clusters.

In this paper, Fig 13 is analyzed from different perspectives. First, compare the distances of *a*_1_ and *a*_2_ to *J*_1_. It can be seen that the ED from *a*_1_ to *J*_1_ is shorter than *a*_2_ to *J*_1_. From the above analysis, *a*_1_ is the leader and *a*_2_ is the follower. From the figure, it can be seen that *a*_1_ gradually approaches *J*_1_. According to the Norm *r*_4_, then *a*_2_ uses the goal following approach toward *a*_1_. In this figure, *a*_2_ by multiple sensors can observe *a*_1_ when *a*_1_ has traveled to *A*. Meanwhile, *a*_2_ has traveled to *A*′. According to the Norm *r*_25_ ∼*r*_27_, so *a*_2_ takes a goal following towards *a*_1_. Until *a*_2_ by multiple sensors do not observe *a*_1_, at this point *a*_1_ travels to *B*. Meanwhile, *a*_2_ has traveled to *B*′. Since both *a*_1_ and *a*_2_ are in the execution task area and *a*_2_ by multiple sensors do not observe *a*_1_. According to Norm *r*_5_, then *a*_2_ takes a trajectory following towards *a*_1_. *a*_2_ travels from *B*′ to *C*′. Similarly, *a*_2_ by multiple sensors to observe *a*_1_ when *a*_1_ has traveled to *C*. Meanwhile, *a*_2_ has traveled to *C*′. According to the Norm *r*_25_∼*r*_27_, so *a*_2_ takes a goal following towards *a*_1_. Until *a*_2_ by multiple sensors do not observe *a*_1_, at which point *a*_1_ travels to *D*. *a*_2_ has traveled to *D*′. Since both *a*_1_ and *a*_2_ are in the execution task area and *a*_2_ by multiple sensors do not observe *a*_1_. According to Norm *r*_5_, then *a*_2_ takes a trajectory following towards *a*_1_. *a*_2_ starts traveling from *D*′. Until both *a*_1_ and *a*_2_ complete their firefighting tasks.

#### Evaluation

This paper statistics the raw data acquired by GBBopen. GBBopen receives the number of localization coordinates of all sensors simultaneously is 4070. The number of coordinates of UWB localization that are larger than the distance to the ground truth is 3608. So FER is calculated as 11.35%.

Table 10 shows compare results of the range of error and MSE between single sensor and multi-sensor fusion. The range of error of ED between the single sensor observation and the true value is 0.80∼1.47 m. The error range of ED between the observed and true values after multi-sensor fusion is 0.59∼1.02 m. The Min of the error of the ED after multi-sensor fusion increased by 26.25% over the single-sensor observations. The Max of the error of the ED after multi-sensor fusion decreased by 30.61% over the single-sensor observations. The range of MSE between the single sensor and real values of the *a*_1_ after multi-sensor fusion is 1.06∼1.35*m*^2^. The MSE of the multi-sensor fusion results over the real value of *a*_1_ is 0.66∼0.86*m*^2^. The Min of the MSE after multi-sensor fusion is 37.74% higher than that of the single-sensor observations. The Max of the MSE after multi-sensor fusion is increased by 36.30% compared to that of the single-sensor observation.

**Table 10:** Compare results between single sensor and multi-sensor fusion.

	Single Sensor	Multi-sensor Fusion
Min Error (m)	0.80	0.59
Max Error (m)	1.47	1.02
Min MSE (*m*^2^)	1.06	0.66
Max MSE (*m*^2^)	1.35	0.86

The analysis of the above experiment results shows that the fusion of multiple sensors is achieved in this paper. This fusion module ensures precise positioning between adjacent unmanned vehicles using multiple sensors. The unmanned vehicles are coordinated to accomplish the firefighting task. This fusion module reduces the impact of FERD system in open environments. This module also improves the environmental awareness of each unmanned vehicle.

## Discussion

Norms are designed by experts in multiple fields. Each expert can describe different fields according to his experience. This fusion module involves multiple domains. Therefore, experts construct complex fusion strategies in a data processing module by combining multiple norms. When the measured data is unstable, this fusion module can reflect a certain degree of robustness. For example, if a sensor is not working or information is confusing, the FERD system can continue to perform firefighting tasks. The FERD system demonstrates its scalability when new needs arise. For instance, this paper adds new norms within this fusion module. These norms can meet the needs of subsequent environments, and long-lasting applications.

This fusion module proposed in this paper suffers from certain limitations. This paper discuss the uncertainties and limitations of fusion module in terms of the delay of the data, the suitability of the sensors, and the protection measures of the sensors.

- **Data latency**. The data acquisition is performed in FERD system with ROS as the medium. The first consideration is how to ensure real-time data fusion. The involved delay includes transmission delay, propagation delay, processing delay, etc. So it is more demanding on the network. FERD system is an event-trigger mechanism. It is more important the specific events monitored change at any time. This mechanism has strong random characteristics. This mechanism cannot predict the target object. In some respects, this may limit the open system. However, in a dynamic environment, this is more generic for fusion methods. This generalization is the fusion of data by norms.
- **Sensor selection**. Fire environments have special properties, such as high temperatures and dense smoke. These special properties can have an impact on the performance and reliability of the sensor. The range of applications for a sensor can also affect its performance and reliability in a fire environment. Namely, some sensors may only be suitable for specific types of fires and may not work properly for other types of fires. Therefore, the range of application of sensors needs to be considered when applying them to avoid limitations and misjudgments. The sensors covered in this paper are a range of DJI-Innovations RoboMaster (EP) products, including an infrared depth sensor (IDS) and a camera. IDS has an operating temperature range of -10 to 40 degrees Celsius. It is possible for the range accuracy to be reduced or even for the measurement to fail in special weather or environments, such as dense smoke, rain, fog, or direct sunlight. The camera needs to ensure that it is not disturbed and is unobstructed. There are video streams and images in the thick smoke. Its operating temperature range is -10 to 40 degrees Celsius. Extreme environments are not considered in this paper. For instance, the fire environment when studying evacuation strategies for large public buildings [25]. Therefore, the sensors involved cannot face complex high-temperature or dense smoke environments and are only suitable for mild fire situations. This paper also proposes some methods to improve the data collection capability of the FERD system and ultimately improve fusion efficiency. For example, sensors that can resist harsh environments could replace the above sensors to further improve their performance and stability.
- **Sensor protection**. Protective measures for sensors in a fire environment are also very important. For example, packaging and encapsulation with fire-resistant materials are required to protect the sensors from high temperatures and smoke. Moreover, the protection level of the sensor and the match between the protection level and the application environment need to be considered to ensure the performance and reliability of the sensor.

At present, many possible applications are being considered to test the feasibility of this fusion module. To move from the current system to the actual application, it is also necessary to add some functions reflecting the real situation in these norms (e.g. the important parameters of some norms). In theory, these parameters use constant values. In application, these parameters use empirical values. These experience values are dynamically adjustable. Also, the parameters can be affected by random, system noise, due to larger environmental factors.

## Conclusion

This paper proposes a fusion module in the FERD system based on Blackboard Architecture. The main goal of this fusion module is to overcome the inaccuracy of a single sensor in indoor scenarios with multiple obstacles and improve measurement accuracy. The module utilizes multiple sensors to complement or correct the positioning of each unmanned vehicle. Specifically, this module develops a variety of fusion-related processing techniques for precise positioning. This paper then proceeds to gain experience data on the confidence degree, error of different sensors, and timeliness of this module by training in an indoor scenario with multiple obstacles. Compared with a single sensor, the module proposed in this paper is switchable and scalable. Finally, the performance of the fusion module was evaluated to demonstrate the effectiveness of localization based on the field sensors. Some important applications are target identification and target tracking tasks for multiple agents. These areas mainly include identifying the motion intention of the target, local path planning, etc. For example, when a sensor has short-term abnormal data, the system determines the abnormal conditions based on the status of the abnormal data and automatically filters this abnormal data. Then this system can still work normally without affecting subsequent operations. In the future, we will apply the proposed fusion module to other applications. The application of autonomous driving systems, for example, can improve safety.

## Supporting information

S1 Data (ZIP)
